# Effects of Multiple Local Environments on Electron Energy Loss Spectra of Epitaxial Perovskite Interfaces

**DOI:** 10.1021/acs.jpcc.2c06879

**Published:** 2022-12-08

**Authors:** Robert A. Lawrence, Quentin M. Ramasse, Kristina M. Holsgrove, Daniel Sando, Claudio Cazorla, Nagarajan Valanoor, Miryam A. Arredondo

**Affiliations:** † Department of Physics, University of York, Heslington, North YorkshireYO10 5DD, United Kingdom; ‡ SuperSTEM Laboratory, SciTech Daresbury Campus, DaresburyWA4 4AD, United Kingdom; § School of Chemical and Process Engineering and School of Physics and Astronomy, University of Leeds, LeedsLS2 9JT, United Kingdom; ∥ School of Mathematics and Physics, Queen’s University Belfast, BelfastBT7 1NN, Northern Ireland, United Kingdom; ⊥ School of Physical and Chemical Sciences, University of Canterbury, ChristChurch8140, New Zealand; # Departament de Fisica, Universitat Politecnica de Catalunya, BarcelonaE-08034, Catalonia, Spain; ∇ School of Materials Science and Engineering, University of New South Wales, Sydney, NSW2052, Australia

## Abstract

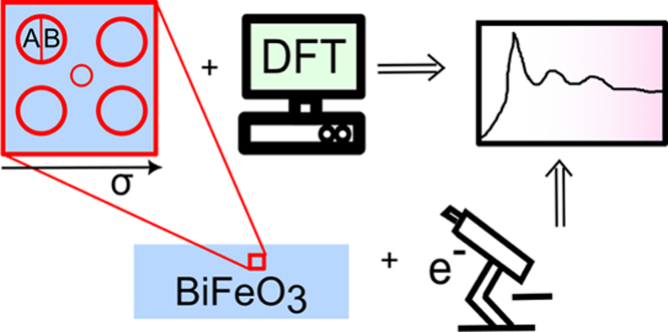

The role of local chemical environments in the electron energy loss spectra of complex multiferroic oxides was studied using computational and experimental techniques. The evolution of the O K-edge across an interface between bismuth ferrite (BFO) and lanthanum strontium manganate (LSMO) was considered through spectral averaging over crystallographically equivalent positions to capture the periodicity of the local O environments. Computational techniques were used to investigate the contribution of individual atomic environments to the overall spectrum, and the role of doping and strain was considered. Chemical variation, even at the low level, was found to have a major impact on the spectral features, whereas strain only induced a small chemical shift to the edge onset energy. Through a combination of these methods, it was possible to explain experimentally observed effects such as spectral flattening near the interface as the combination of spectral responses from multiple local atomic environments.

## 1 Introduction

Multiferroics are materials that exhibit more than one ferroic order in the same phase, resulting, for example, in coupling between strain and electric fields (piezoelectrics) or between magnetic and electric fields (magnetoelectric) within the material. In their different forms, this class of materials constitutes the basis for many current technologies including a variety of actuators, sensors, and memory devices. These materials continue to be extensively studied due to their potential, especially as thin films, for use in novel applications.^1,2^

Interestingly, effects such as diffusion,^3^ point defects (including vacancies),^4,5^ and strain gradients^6−9^ are now deliberately introduced to modify the electronic structure and induce (and tune) new functionalities in multiferroic thin films such as electrochemically induced strain^10^ and oxygen-vacancy-controlled magnetism.^11^

The development of such engineered multiferroic thin films, as well as other novel functional interfaces,^12^ has relied on their characterization by electron microscopy techniques,^13−15^ in particular, through the combination of scanning transmission electron microscopy (STEM) and electron energy loss spectroscopy (EELS), where unprecedented spatial and energy resolution can now be achieved (<1 Å and 15 meV).^16^ Core-loss EELS is highly sensitive to local crystallographic, chemical, and electronic structural changes, while low-loss EELS can provide information on the optical response and band gap of materials.^17^ Thus, STEM–EELS has been instrumental in providing key insights into the relationship between the structure and the properties of what can be rather complex functional systems such as multiferroic oxides.^18^

Core-loss EELS has been extensively used to obtain information on the oxidation state,^19,20^ coordination,^21^ octahedral tilts,^22^ and the presence of vacancies in multiferroic thin films^23^ by analyzing the fine spectral features of the electron energy loss near-edge structure (ELNES) of both oxygen and metal ions. These spectral features arise due to the transition of electrons from a core orbital to unoccupied bands, reflecting the partial density of states and hence enabling a direct fingerprinting of the material’s structure and chemical environment.^24^ Thus, the information contained in the ELNES fine structures is directly related to the electronic structure of the material and ultimately to the material’s properties. Therefore, understanding (and correctly interpreting) the ELNES fingerprint can help us to answer some of the currently unresolved questions regarding the origin of functionality in fields such as materials science and catalysis where complex functional materials are being developed. The technique is particularly powerful for investigating effects and functionality, which originate at the nanoscale, such as interfaces, where changes in bonding and oxidation state occur over small distances. Current STEM–EELS instrumentation routinely allows for tens of spectra to be acquired as a “hyperspectral data set”, or spectrum image, across a single area of interest with atomic precision, providing a wealth of information. However, such vast quantities of information and the subtle differences that can be found in the ELNES features mean that a full, direct, and clear interpretation of the physical mechanisms at play based on the experiment alone can be challenging, if not impossible. This means theoretical calculations are crucial to fully interpret the information contained in the EEL spectra. In recent years, significant efforts have been directed at advancing the theoretical methods for calculating ELNES, such as the inclusion of the core hole effect.^25^ Furthermore, Tomita et al.^26^ have significantly improved the theoretical method by including many-body effects, which enabled them to successfully reproduce the experimental O K-edge of perfect bulk perovskites.

However, the advances in EELS calculations rarely combine experiments with calculations in a systematic fashion, especially for more complex systems. To date, a more comprehensive theoretical framework exploring how the interplay of effects found in engineered multiferroic thin films (diffusion, strain, vacancies, etc.) alters the ELNES signature is missing. Resolving this—even in part—requires (i) the use of a supercell with hundreds of atoms, both so that the dopant concentration can be well represented and, importantly, to accurately model the different possible environments; (ii) the simulation of hundreds of spectra to probe each of the multiple unique chemical environments that can be found in the sample (the challenge here lies in the large simulation cells that significantly increase the computation time and push the size of the system beyond what is tractable for a many-body approach); and (iii) simulating the role of probe propagation on the recorded spectra through the use of additional techniques such as multislice simulation.^27,28^

In this work, which focuses on addressing some of the questions arising from requirement (ii), an equivalent of the density functional method within the generalized gradient approximation (GGA) used by Tomita et al.^26^ has been used to simulate the O K-edge in a variety of local—structural and chemical—environments. This level of theory was found to be sufficient to capture the key features of pure, single-crystal systems such as BaTiO_3_ at a qualitative level. While there is some sacrifice in the accuracy of peak intensities and energies compared with their many-body approach, the trends in the ELNES features can still be reliably accessed with a significantly lower computational cost; this is arguably a necessary compromise when dealing with the multiple chemical environments found in real complex functional oxides.

It should be noted that the work here presented is not concerned with perfectly reproducing the experimental spectrum, as many computational and experimental factors can affect these. However, the focus is to demonstrate that it is possible to identify trends that qualitatively match experimental and calculated spectra, using BiFeO_3_ as our prototypical example. These trends may then be used to identify possible physical mechanisms responsible for spectral signatures that result from adding perturbations to the structure such as doping and strain.

It is also worth recalling the well-known fact^29,30^ that errors in differences between density functional theory (DFT) calculations are much smaller than those for a single calculation. This means that even when the experimental spectrum is not perfectly reproduced, useful conclusions about the effects of perturbations to the structure such as applied doping and strain may still be made.

Given its great potential and current relevance in today’s research in the field of functional oxides,^31,32^ we use BiFeO_3_ (BFO) as a model case to investigate the effects that applied in-plane strain and doping or chemical substitution (arising, e.g., from diffusion from a growth substrate or electrode) have on the O K-edge ELNES of this system. In the following work, the “dopant” atoms considered are Mn and La, simulating widely observed interdiffusion across a La_1–*x*_Sr_*x*_MnO_3_ (LSMO) interface, commonly used as a bottom electrode for BFO thin films.^23,33,34^ The same interface is also studied experimentally in Section 4.6 to illustrate a number of features observed in the theoretical calculations.

By using several small supercells to capture fixed strain and chemical composition, which may be considered broadly representative of different regions near the BFO/LSMO interface, it is possible to reduce a region of material that displays a great degree of chemical complexity to simpler models. This enables a more confident link to be made between the structural changes observed in the models and the spectral features these models predict. This use of doped supercells, and the higher-energy resolution of our calculated (and experimental) spectra, goes beyond previous efforts in the field^35^ and will enable conclusions to be drawn about finer features that are now experimentally accessible.

In this work, we will use a combination of computational and experimental techniques to investigate both the atomistic mechanisms responsible for the spectral changes in the O K-edge of BFO—including a discussion on the effects at and near an interface with LSMO. Particular attention will be paid to analyzing the effect that the different local chemical environments have on their spectra and the ability to resolve these experimentally.

## 2 Computational Methodology

### 2.1 Geometry Optimization

Geometry optimization was performed within the VASP code^36−39^ using projector-augmented wave (PAW) pseudopotentials and the Perdew–Burke–Ernzerhof (PBE) functional.^40^ The rhombohedral phase of BFO (space group *R*3*c*) was selected as the starting structure for our calculations, as it is the ground-state configuration of BFO.^41^ A BFO supercell containing 20 atoms was used to reproduce the polar nature of the system and the antiferrodistortive configuration of the O octahedra. A single La ion and either 1 or 2 Mn ions were introduced into the BFO structure to dope the supercells. To select the position of the Mn dopant atoms, all possible configurations were generated by inserting Mn into the bulk rhombohedral structure before being relaxed, with the lowest-energy (most stable) configuration being selected. A Hubbard-U correction of 4 eV^42^ was used for the Fe and Mn 3d-orbitals to account for the strong correlation effects that electrons in these orbitals experience. Wave functions were converged to 1 × 10^–6^ eV/atom, and forces were converged to 1 × 10^–2^ eV/Å. An energy cutoff of 600 eV was used with an 8 × 8 × 8 Monkhorst–Pack grid. In our simulations, epitaxial strain was reproduced by fixing the length of the *b*/*c* in-plane lattice parameters to render deformations of −0.20, 0.85, 1.88, and 2.94% with respect to the unconstrained theoretical system (which covers a typical range of strains found near the interface of epitaxial thin film perovskite oxides^43^). It should be noted that the strain values here used differed from integer percentages (0, 1, 2, 3%) typically reported due to the slight differences in the ground-state calculation when the geometries were transferred into the CASTEP code. The differences are, however, small, and the strains remain within typical values for BFO systems. The authors would like to emphasize that since strained systems are being deliberately studied, the fact that the ground-state strain is not exactly found does not affect the conclusions of this work. The out-of-plane lattice vector was subsequently fully relaxed along with the atomic positions.

### 2.2 Simulation of Spectra

The CASTEP code^44^ was used to perform simulations of the EEL spectra for each oxygen atom in the supercell, through the calculation of transition dipole matrix elements and the application of Fermi’s golden rule. The PBE functional^40^ was used with a plane-wave cutoff of 700 eV and a Γ-centered Monkhorst–Pack grid of 10 × 10 × 10 *k*-points.^45^ The total SCF energy of the system was converged to 1 × 10^–6^ eV. Three hundred extra bands were used to simulate the unoccupied energy levels up to at least 30 eV above the onset energy. To simulate the overlap of the core orbitals with the valence orbitals, and thereby accurately calculate the transition dipole moments, Blöchl’s PAW method was used to reconstruct the wave function within the pseudisation radius of the pseudopotentials.^46^ All spectra were normalized over all strain and doping cases to the tallest peak observed in this series of calculations (the A-peak of O7 in the 1.92% strain case of 50% Mn-doped BFO) to enable direct comparison between cases. Composite spectra were generated by an even weighting of all prenormalized spectra (so dividing through by the number of contributing atoms was the only additional step). With fixed geometry, varying the Hubbard-*U* value had no effect on the spectra (see Figure S1) since it only directly affects the d-density, which was not sampled in the spectra due to dipole selection rules for a transition starting in a state with s-symmetry.

The Slater transition-state model, corresponding to the excitation of half an electron from the core level to the valence band, was chosen to represent the core hole,^47^ a technique used successfully by Naz et al. for α-BFO.^48^ This has the advantage over the initial state rule,^49^ in that it simulates the experimentally observed excited state directly. It also has an advantage over the final state rule,^50^ which produces results that tend to be underscreened, particularly in the case of semiconductors. This also makes sense for the system when the rule of thumb of Mauchamp et al.^51^ is considered. This rule suggests that the core hole is important for atoms with a significant density at the conduction band edge. For BFO, there is a moderate presence of the O-related density of states at the conduction band edge, suggesting a weaker core hole effect. This is a further reason for selecting the Slater transition state rather than a full core hole. The choice of placing the extra electron directly into the valence orbital ensures that the excitation is electrically neutral, so the self-interaction of the core hole with itself is minimized, making the 20-atom supercells sufficient.

In addition to calculating the spectra, the edge onset energies were calculated using the method of Mizoguchi et al.,^52^ which enables the calculation of an excitation energy to within a few percent with a pseudopotential code.^53^ This method is preferred over a more simple approach relying on fitting the edge onset to experimental data, as it includes atom-scale detail, which would otherwise be ignored if a simple rigid shift to the experimental onset had been used instead, thereby enabling a self-consistent comparison with the experiment.

**Figure 1:**
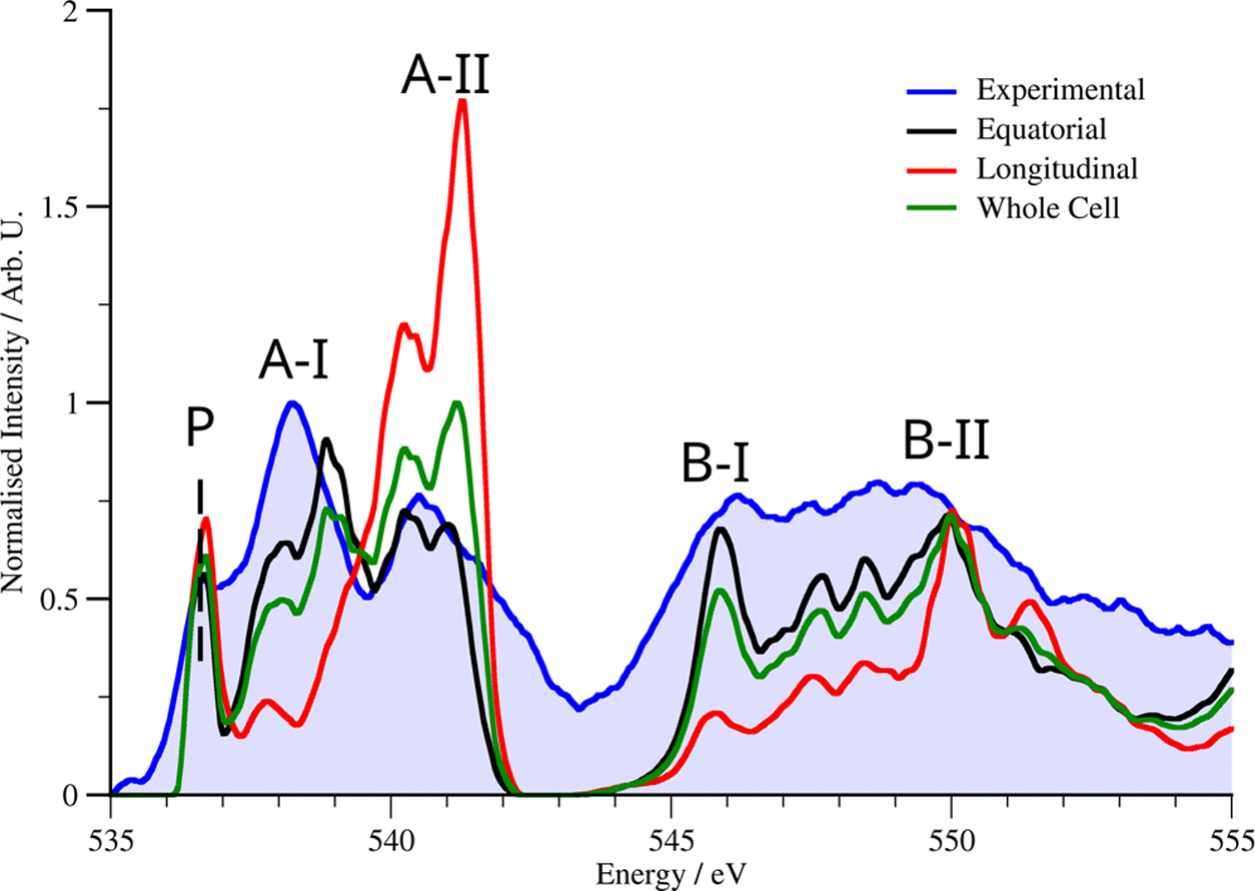
Simulated and experimental O K-edge for BFO along the [001] direction. A fixed Gaussian broadening of 0.12 eV was used for the simulated spectra. The simulated spectra have been grouped according to an equal weighting over a single unit cell; when the beam propagates along the *b⃗* direction, this leads to the columns consisting of FeO (longitudinal environment) or just of O (equatorial environment)—see Figure 2. All simulated spectra were normalized against the whole unit cell average.

For simplicity and to reduce computational cost, only one incoming plane wave was considered for the simulations—the probe convergence angle thus not being explicitly considered. The spectra were calculated with an incoming wave polarized along a [111] direction (apart from Figure 1 where the beam was simulated along the [001] direction), which allows a study of the effect of structure and chemical composition on the fine structure, as projected along all spatial directions. The calculated spectra contain all components—*x*, *y*, and *z*—with none being suppressed due to selection rules, providing additional insight (by capturing the effects of chemistry along all three axes) at the cost of a less direct comparison to experimental data obtained along different zone axes of the structure.

To a good approximation, dipole-allowed transitions dominate experimental EEL spectra. Higher-order contributions are allowed; however, they should be minor because the Rutherford cross section (appropriate to the scattering of charged particles, as is the case in EELS) reduces the contribution from scattering events with large *q*.^54^ The influence of these large *q* events may also be reduced experimentally by reducing the axial collection angle.^46^ Conversely, experimental channeling effects change the beam from a plane wave to something more locally focused—containing many different electron momenta^55^—which could be expected to enhance the contribution of nondipole terms. Once again, in the interest of balancing computational tractability and scope, the dipole approximation has been used in this work.

The calculated spectra do not take into account effects such as channeling through the sample thickness before and after the inelastic event, which are known to affect the experimentally observed fine structure. These effects can be simulated by combining DFT-based spectral calculations and multislice beam propagation algorithms^27,56^ but at great computational cost. The multitude of parameters considered here and the large simulation cells that this would require for a full beam propagation calculation in each case would make such an approach impractical with currently available computational tools. However, by understanding the chemical and structural origins of changes in the atomic spectra, we can gain precious insight into changes observed in experimentally acquired spectra.

To take into account the various broadening effects and to facilitate a comparison with experimental results, an *ad hoc* Gaussian broadening of either 0.1 and 0.5 eV (for pure and impure systems, respectively) was applied to the spectra. This was a fixed broadening (not varying dependent on the energy of any point in the spectrum) and as such may be considered to include mechanisms such as phonon (thermal) broadening but does not include any explicit lifetime broadening—which is dependent on both the energy of the state above the Fermi level and its character. However, this does not affect the qualitative interpretation of the results (focusing on the relative intensity and peak positions), which is the main aim of this work. To get the lifetime broadening in an *ab initio* manner would also require the use of a more computationally intensive theory than used in this work, such as time-dependent density functional theory (TDDFT). It may also be noted that using a relatively large broadening for the doped systems helps avoid overinterpretation of the results, as this will remove features that appear due to numerical noise.

## 3 Experimental Methodology

### 3.1 Sample Growth

The BFO and LSMO films were grown on LaAlO_3_ (001) substrates by pulsed laser deposition.^57,58^ The LSMO layer was grown at a substrate temperature of 750 °C, with a 100 mTorr oxygen pressure, and a laser fluence of ∼3 J/cm^2^. Next, the BFO layer was grown at a substrate temperature of 590 °C and a background oxygen pressure of 100 mTorr. The plume was generated using a 248 nm KrF excimer laser at 10 Hz, with fluence at the target of ∼1.8 J/cm^2^. After growth, the samples were cooled to room temperature at 5 °C/min in 5 Torr of oxygen. Samples were prepared for electron microscopy observation using a focused ion beam instrument using conventional thinning procedures.^59^

### 3.2 Spectra Acquisition

STEM–EELS and simultaneous annular dark-field (ADF) imaging data were acquired on a Nion UltraSTEM 100MC “Hermes” microscope equipped with a cold field emission electron source and a high-resolution ground potential electron beam monochromator. The microscope was operated at 60 kV to maximize the EELS experimental cross sections and to minimize the risks of knock-on damage to the sample by the electron beam. The probe-forming optics were adjusted for a convergence semiangle of 31 mrad resulting in an estimated probe size of ∼1 Å. Unless otherwise specified, the monochromator slit was positioned to provide an energy resolution of 100 meV, as estimated from the full width at half-maximum (FWHM) of the zero-loss peak (ZLP), a compromise between the optimal resolution achievable with the system, the intrinsic width of spectral features in ELNES of the O K-edge (limited by effects such as finite lifetime broadening), and the probe current and signal to noise. The EELS data were recorded using a Gatan Enfinium ERS spectrometer optimized with high-stability power supplies, with a spectrometer entrance aperture of 44 mrad semiangle. The high-angle ADF (HAADF) detector angular range was 92–195 mrad, while a medium-angle ADF (MAADF) detector with a 55–92 mrad angular range was also used.

## 4 Results and Discussion

### 4.1 Computational Results

It is known that even at atomic resolution, the final spectrum is the result of a complex combined contribution of signals from all atoms along the electron beam path. In principle, one can imagine that in a carefully grown heteroepitaxial thin film, all equivalent atoms in each column would have a similar local environment with respect to neighboring atoms, including tilt, and strain. In reality, localized defects and chemical inhomogeneities can be present (even deliberately introduced) and these will affect the ELNES features. Thus, when it comes to modeling, giving equal weight to all of the atoms does not necessarily give a faithful representation of the material under investigation.^27^

This is especially important when making comparisons to the experimental spectra since for a small probe size (<0.4 nm, the size of the unit cell), not all of the atoms in a unit cell will be sampled equally, and indeed channeling effects will mean that the probe beam will sample nearby columns of atoms. This is significant because, as discussed in Section 4.6, even within a chemically homogeneous material, it is possible to find multiple local environments, which cause subtle but significant changes to the spectral features. To fully reproduce an experimental spectrum, it would be necessary to model the channeling effects to determine how the probe combines the signals from separate atoms.

These calculations are not readily tractable for complex supercells, however, due to the loss of symmetry. Therefore, here we have simulated the spectra “as electrons leave the atom” and do not consider the weighted combinations directly. By examining the contributions from individual atoms within the *R*-phase BFO crystal structure, and how they are affected by subtle chemical and structural changes, it is still possible to identify trends that can then be used to gain insight into the physical origin of the features found in the experimental spectra, even though spectral matching remains beyond the scope of this work.

Previous experimental work on BFO/LSMO interfaces has found that the O K-edge spectra adopt a very different structure at the interface compared to deep inside the individual layers,^60^ frequently becoming a single broad feature rather than a series of well-defined peaks. Several origins for this “spectral flattening” have been considered, including doping effects, channeling, and O vacancies; however, the cause of this feature remains unclear.

A typical oxygen K-edge for *R*-phase BFO comprises a sharp peak A (∼530 eV), a peak B (∼542–555 eV), and a broad section (above ∼560 eV) where further peaks C and D can be found (see Figure 1). All of the peaks come from the hybridization of the O 2p valence orbitals with orbitals from the cations. The origin of the A- and B-peaks is well established.^5,61,62^ Peak A arises from the interaction between O 2p and the A-cation: Bi 6p and La 5d orbitals for the doped case; peak B originates from the hybridization with the 3s and 3p orbitals of the B-site cation (Fe or Mn when considering a doped system), which leads to its characteristic splitting. The origins of peaks C and D are less clear, as they appear to be extended energy loss fine structure (EXELFS) rather than ELNES,^63^ and they are not commonly discussed in the wider literature due to the difficulties in resolving them experimentally. Additionally, in this work, we will refer to a prepeak (P), found before peak A, which arises due to the p-like character of the O 2p bonding with the B-site 3d orbitals.

Figure 1 illustrates these peak assignments through a comparison between an average high-energy-resolution experimental spectrum (see Section 4.6) and simulated spectra for pure *R*-phase BFO using the methodology described above. These spectra are split according to the local environment of the O atoms with respect to the Jahn–Teller distortion of the octahedra, as well as being average over the entire unit cell. These new spectra (both experimental and the spectrum for the equatorial environment) agree well with prior comparisons between the experiment and theory (see Rossell et al.^64^), albeit taking advantage of the higher-energy resolution available in modern instruments. Experimental spectra with wider energy ranges comprising peaks C and D can be found in Figure S2.

**Figure 2:**
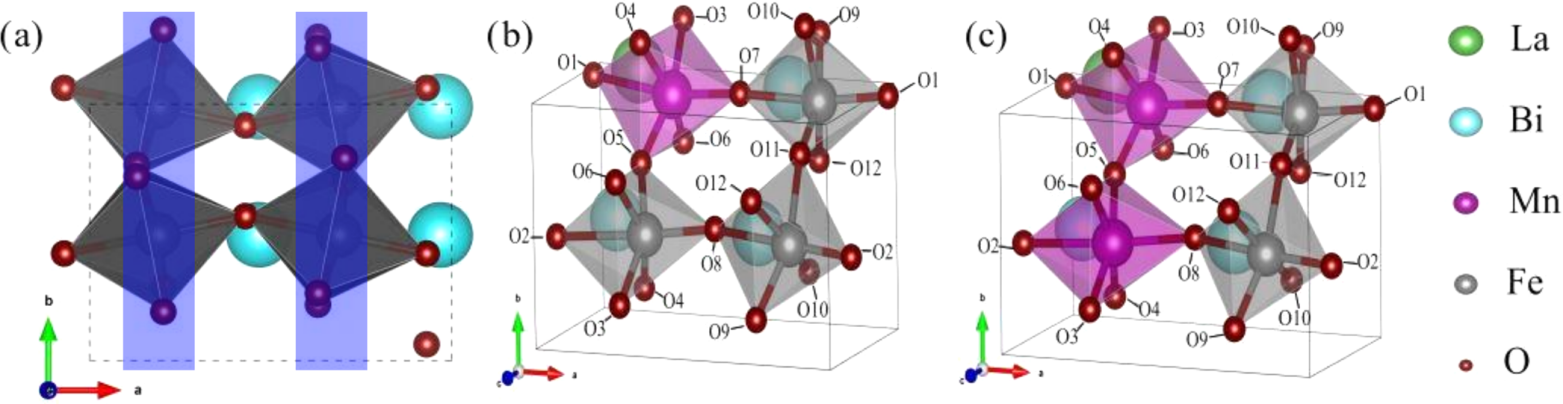
Simulated unit cells used in this work. (a) An undoped bulk-like rhombohedral (*R*)-phase BFO structure. Blue boxes indicate the O atoms (red spheres) considered to be in “equatorial” environments, whereas the other O atoms are in “longitudinal” environments, (b) the 25% Mn, 25% La, and 50% Mn BFO systems, and (c) the 25% La-doped BFO system. The oxygen ions are labeled according to their position and environment: (i) O3–O6 are designated as the “equatorial near Mn” environment, (ii) O9–O12 are in the “equatorial not-near Mn” environment, (iii) O1 and O2 are in the “nonequatorial near La” environment, and (iv) O7 and O8 are in the “nonequatorial not-near La” environment. For clarity, O ions outside of the unit cell are shown, which means more than one periodic image of certain O ions are present. As expected, the applied in-plane strain stretches the *b* and *c* lattice vectors and simultaneously shrinks the lattice vector.

It is worth noting that in Figure 1, there is a significant difference in the match with the experiment for the equatorial environment (which qualitatively correctly reproduces all of the features of the experimental spectrum) and the longitudinal environment—and due to this the unweighted average of the whole unit cell. For the identity of the atoms in each environment, see Figure 2. Since all spectra come from the same model, it seems implausible that somehow one environment can be correct and the other wrong, suggesting a physical mechanism—such as convergence and collection effects or channeling—that suppresses the signal from atoms in the longitudinal environment.

In a thin sample, the majority of the beam will adhere to the local column it enters at the surface (this is known as channeling).^65^ When the beam propagates along the [001] direction, this leads to the *b⃗* columns consisting of FeO (longitudinal environment) or just of O (equatorial environment). Naively—and unjustifiably—assuming equal scattering probabilities for Fe and O, this would instantly lead to a reduction by a factor of 2 for the intensity of the O signal from these atoms. However, the probability of scattering from an Fe and an O atom is not equal, with the Rutherford cross-sectional contribution to the probability having an approximately *Z*^2^ dependence^66^ (where *Z* is the atomic number). This alone gives a scattering rate from Fe atoms that is in the order of 10 times greater than that for O, leading to a significant suppression of the signal from atoms in the longitudinal environment. A detailed evaluation of these effects could be made using a multislice simulation.

Throughout this section, selected oxygen atoms, which are representative of the different local environments, have been chosen to illustrate the trends in the variation of the O K-edge ELNES as a function of doping and strain. The two most significant parameters used to identify the groups were their Jahn–Teller environment (either equatorial for the four closer oxygen atoms or longitudinal for the two further away oxygen atoms) and the proximity of these atoms to any dopants (La or Mn). Even within these basic environments, a small degree of variation was observed between spectra since the octahedral tilts meant that different bond lengths and angles were present, further reducing the symmetry.

The averaged spectra for different in-plane strains for the doped systems can be found in Figure S2. Only minor variations in the spectra were found with the varying strain, suggesting that the BFO is robust to the applied strain. Then, doped and strained supercells were used to simulate different chemical environments, which might be found near a BFO/LSMO interface. The relaxed simulated crystal structures are shown in Figure 2. Panel (a) shows pure BFO, panel (b) shows La_0.25_Bi_0.75_Fe_0.75_Mn_0.25_O_3_, and panel (c) shows La_0.25_Bi_0.75_Fe_0.5_Mn_0.5_O_3_. For each oxygen, individual K-edges were calculated and then an average of all of the spectra was taken.

The first observation from the simulated spectra for the doped and strained cases is that these followed the broad structure of the undoped bulk-like BFO spectra, with large A- and B-peaks followed by smaller C- and D-peaks.^61^ However, small but significant differences are observed due to the dopants’ presence, which will be discussed in the following sections.

### 4.2 Projected Densities of States (PDOS) of Systems

To gain physical insight into the origin of the peaks for the doped BFO systems, it is important to identify the orbitals that give the most significant contributions to these peaks. This can be done by inspecting the projected densities of states (PDOS) for the pure and doped systems. Moreover, these PDOS can assist with determining whether an experimental feature is genuine or noise. This analysis also clarifies the mechanism for the action/effect of dopants: whether they introduce a new set of orbitals or whether they perturb existing orbitals through electron-withdrawing or donating effects. The PDOS simulated in this work, shown for completeness in Figures S3 and S4, are in broad agreement with previously reported simulation efforts^5,61^ for pure and single-dopant modified BFO.

However, the PDOS alone is not sufficient for accurately replicating or interpreting an experimental spectrum, as it does not take into account selection rules. This is most clearly seen in the neglect of the dipole selection rules, which account for the small contribution made by the d-orbitals to the prepeak, which is due to s → d transitions being dipole-forbidden. For this reason, a more rigorous analysis and calculation of the spectra require a knowledge of the matrix elements, which have been calculated under the dipole approximation in this work.

### 4.3 Onset Energies

A further consideration when calculating the average spectrum of each unit cell is the edge onset energy. The range of calculated onset energies for the edges presented here is in the order of 1 eV for every strain case of every doped system. This chemical shift has been reported before from both a theoretical^67,68^ and experimental^69^ perspective, emphasizing that any manual shift of the edges to a “standard onset” for the element (frequently done by matching a peak to its experimental equivalent) is discarding useful data about chemical shifts.

When these chemical shifts are accounted for by setting the spectra for individual atoms to their own unique onset energy—rather than performing an *ad hoc* shift to the experimentally recorded onset—the main peaks from different atoms may no longer perfectly align. This has the effect that whenever the spectra are combined in this way, the observed peaks of the spectra from multiple atoms will be broader than those recorded for an individual atom in what might be considered to be “chemical shift broadening”.

This additional broadening has not been pursued in previous simulations of EELS, which have usually focused on homogeneous systems where such effects should be minimal. However, in the case of a system with multiple chemical environments, this effect can modify the observed experimental signal.

While there may be a large absolute difference between the onset values here observed and those experimentally recorded, they are within a 2% error interval of the expected experimental result, which is the typical tolerance for the method used to calculate the onsets.^53^ These errors are also expected to be systematic rather than random, so that the chemical shifts between different atoms in the same unit cell are preserved.

**Figure 3:**
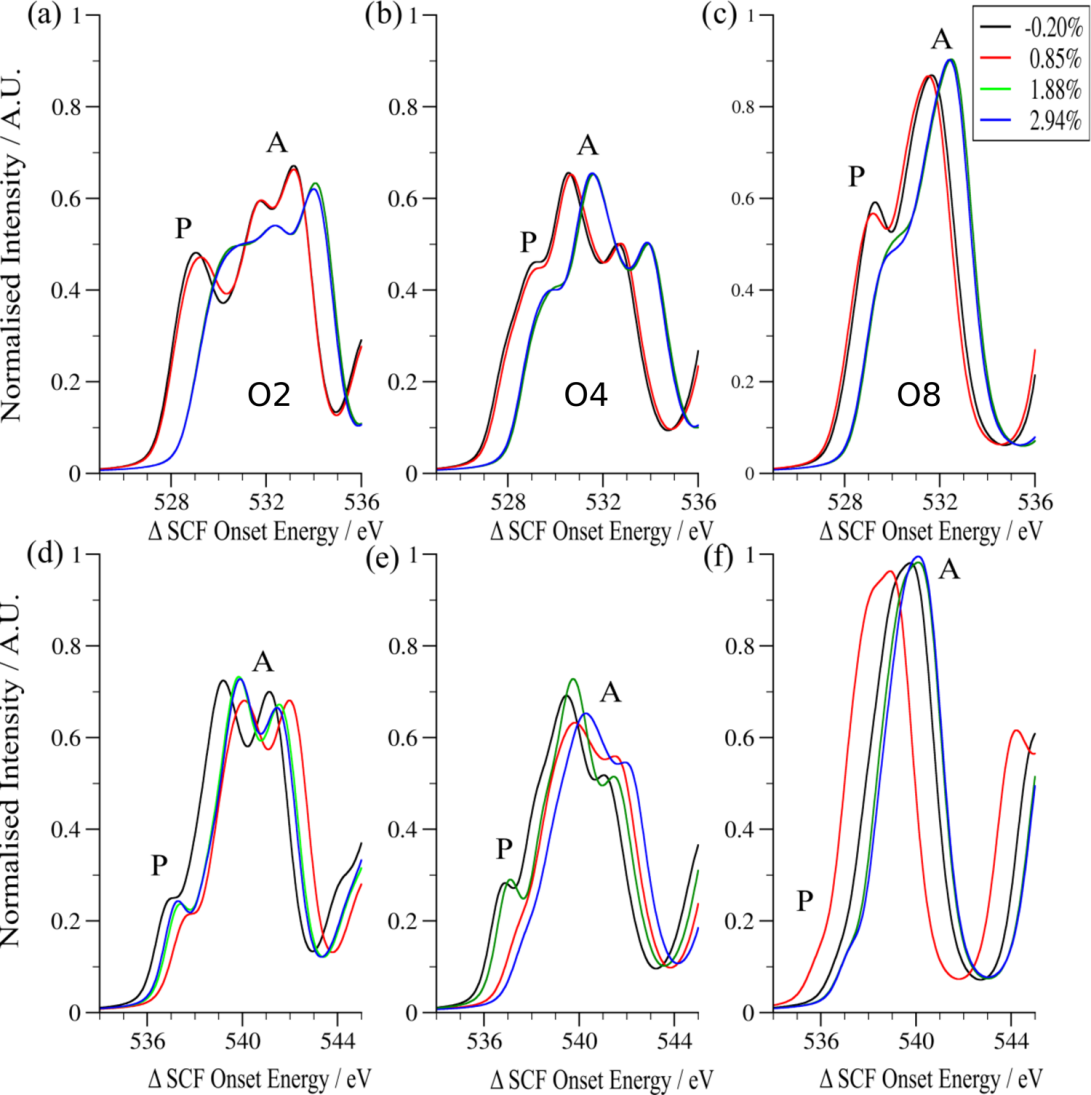
Simulated A- and prepeaks (P) of the 25% Mn-doped case for (a) O2, (b) O4, and (c) O8 and 50% Mn-doped case for (d) O2, (e) O4, and (f) O8. Each O ion is presented at four different strain values. All spectra are normalized against a constant value.

**Figure 4:**
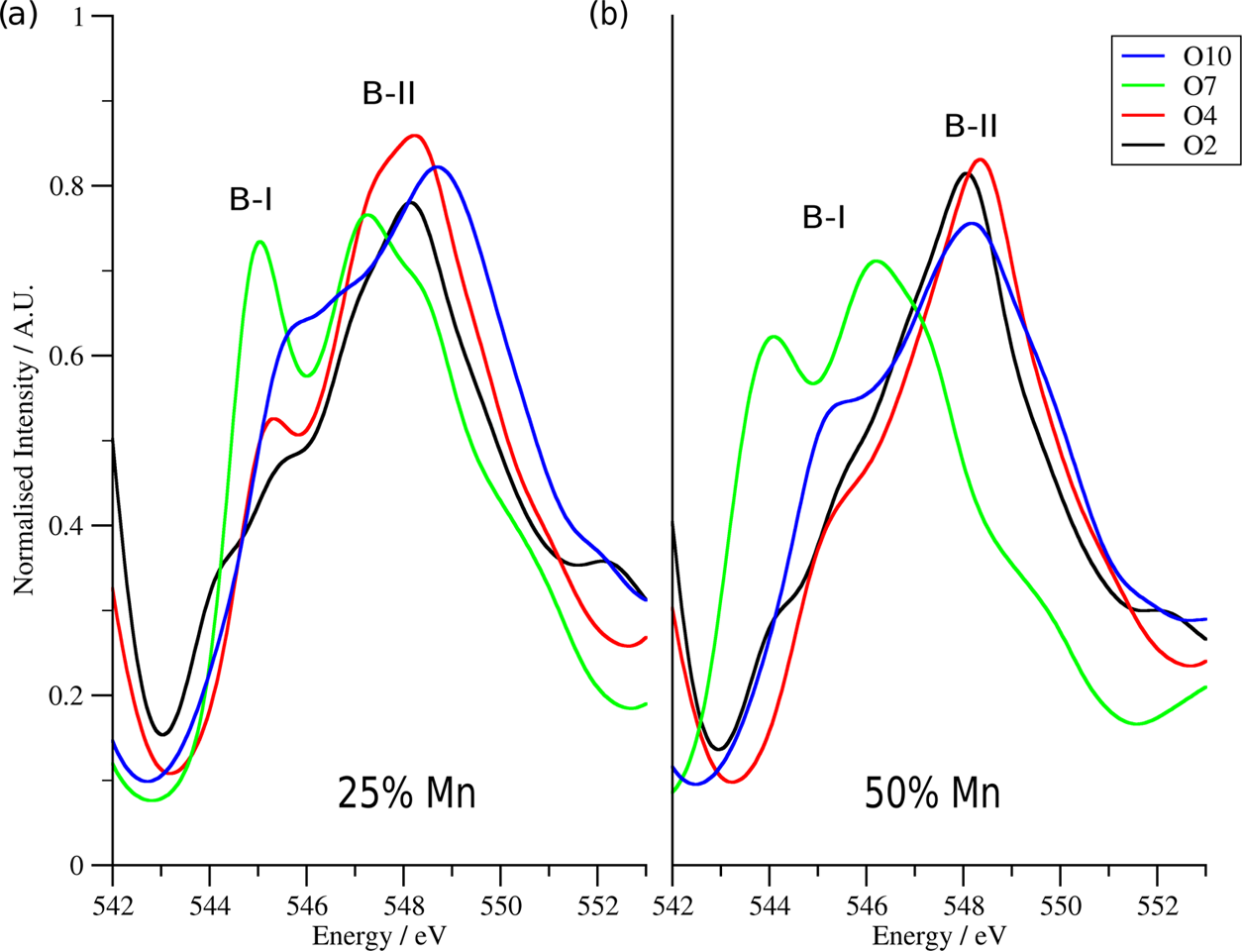
Simulated B-peaks for O2, O4, O7, and O10. The −0.20% strain case is shown. Panel (a) is for the 25% Mn doping case and panel (b) is for the 50% Mn case. The feature to the left of the B-I peak is the tail of the A-peak. Note the relative suppression of the B-I peak with increasing Mn concentration.

In this work, a clear change in absolute energy of the peaks over strain due to the variation in onset energy is observed. In the 25% Mn case, there is a distinct jump of 1 eV in the onset energy between the 0.85% strain case (Figures 3 and 4) and the 1.88% strain case, whereas the 50% Mn system shows a steadier variation with strain, as shown in Figure S2. This change in onset energy can be associated with the corresponding jump in the lattice parameter between the strain cases (see Table 1). However, it is not clear whether it is strain acting directly through the elongation of bonds or strain acting indirectly via octahedral tilt and change of angle that is causing this shift since strain and tilt cannot be uncoupled.

**Table 1:** *a⃗* Lattice Parameters for the Simulation Cells for the Two Doping Cases^a^

strain	–0.20%	0.85%	1.88%	2.94%
25% Mn	8.737 Å	8.605 Å	8.346 Å	8.320 Å
50% Mn	8.690 Å	8.560 Å	8.432 Å	8.324 Å

a The 25% Mn case has a jump in lattice parameter between the 0.85% strain and 1.88% strain cases that is not present in the 50% Mn case.

That a change of lattice parameter should induce a change in the onset energy is unsurprising. The onset energy is the relative difference between the energy of the core state (which should be nearly constant with respect to any change of environment of the atom) and the first unoccupied state into which the electron can be excited. For the periodic systems simulated here, the decreasing out-of-plane lattice parameter leads to an increase in the energy of this orbital, thereby increasing the onset energy. This may conceivably be an effect of considering different strains as isolated snapshots rather than as part of a larger structure with a strain gradient, and therefore further investigation is required. However, it is still worth noting that relative shifts of the onset energy could potentially be used as a way to monitor and probe the change in local strain in complex systems such as multiferroic thin films, provided that the crystal is pure enough that these shifts are not obscured by other effects.

### 4.4 Simulated Prepeak and A-Peak

Figure 3 shows the prepeaks (P) and A-peaks of O2 for both doped BFO systems. Both O2 and O8 are from longitudinal environments; however, O2 is much closer to La (∼2 vs ∼5 Å), and O4 is representative of the equatorial environment containing Mn. The spectra are all normalized against the value of the largest peak in all of the simulations: the A-peak of O7 for the 2.94% strain case of the 50% Mn-doped system. All of the spectra are set to their theoretical onset energies, calculated using the method of Mizoguchi et al.,^52^ as described above.

The prepeak (P) depends on the interaction of the oxygen orbitals with d-orbitals of the B-site cations. This is expressed through the change of the O p-orbitals, as transitions into the empty d-orbitals are dipole-forbidden. On average, the intensity of the prepeak decreases with increasing in-plane strain and is more prominent at a lower B-site dopant concentration (25% Mn). This trend is likely due to the fact that the Mn d-orbitals occur at a higher energy than the Fe d-orbitals (Fe’s higher nuclear charge increases the size of the attractive Coulomb interaction the electrons experience), and the Mn 3d-orbitals occur at the same energy as the A-peak. This causes the response from O atoms bonding with Mn to be obscured, whereas for Fe a distinct, lower-energy peak may be observed. Additionally, there is a clear grouping of the magnitude of the prepeak and A-peak with respect to strain for the 25% Mn case: the spectra of the two lower strain cases having a more intense peak than the two higher strain spectra. This may be attributable to the change in orbital overlap with varying bond lengths causing a reduction in the intensity of the hybridized orbital that forms the prepeak, thereby reducing the available number of final states for the excitation. It is also worth re-emphasizing the discontinuity in the out-of-plane lattice vector in the 25% Mn case, which was not present in the 50% Mn case; this large jump is the origin of the discontinuity of the spectra with respect to strain.

The high degree of localization of the d-orbitals means that the bonding interaction between the d-orbitals and the O p-orbitals is very sensitive to changes in the atom’s local environment. Therefore, considerations such as the degree of polar distortion and the amount of octahedral tilt can have greater contributions to the resulting spectral features than strain alone. This means that the spectra of individual oxygen atoms do not necessarily obey the general trend observed here: a decreasing prepeak intensity with increasing in-plane strain and/or B-site (Mn) doping.

**Figure 5:**
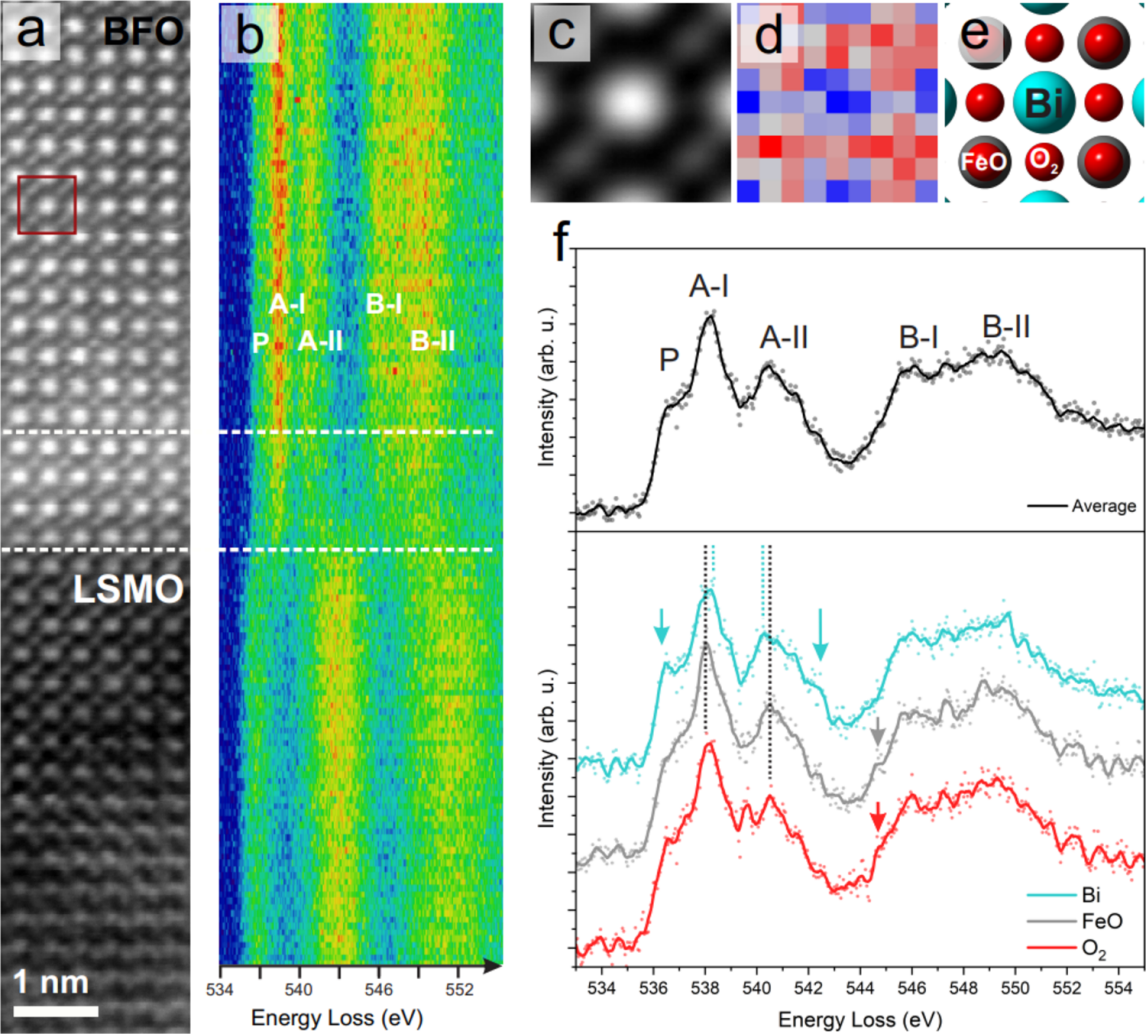
Panel (a) shows the HAADF image of an LSMO/BFO interface. Dashed white lines are guides to the eye, indicating the interface region. A red box indicates a unit cell template, representative of “pure” BFO (far enough away from the interface that chemical interdiffusion is not detected in the EELS data: see Figure S5). This template was used for real-space averaging. Panel (b) shows the EELS spectra for the O K-edge as it develops across the interface, clearly showing the 2-peak structure of LSMO at the bottom, the 3-peak structure of BFO at the top and a flatter region of overlap between them. Panel (c) shows the template-matched average across the pure BFO region, obtained using the red box in panel (a) as the template. Panel (d) shows the (real-space) averaged O K EELS map for pure BFO, obtained by integrating the O K intensity over a 50 eV window above the edge onset after template matching all pure BFO unit cells away from the interface. Panel (f) shows two sets of spectra; the spectrum in the upper panel (black) is the unit cell wide average O K-edge (after template-averaging over the entire pure BFO region), whereas the three spectra in the lower panel are spatially resolved spectra extracted from the template average unit cell from the Bi, FeO, and O_2_ positions in the average unit cell, illustrated in the ball model in panel (e). The cyan (top) spectrum is averaged over the pixels corresponding to the Bi environment, the middle (gray) spectrum corresponds to the FeO (longitudinal) environment, and the bottom (red) spectrum corresponds to the O_2_ (equatorial) environment.

It is also worth noting that the prepeak had been experimentally observed as a shoulder in spectra for the bulk *R*-phase BFO (with an energy resolution of 0.6 eV),^64^ but here it is clearly resolved at a 0.1 eV energy resolution for specific atomic positions in an average BFO unit cell (see Figure 5f).

The A-peak (starting at 532 eV for the 25% Mn case and 536 eV for the 50% Mn case) is in most cases split into two subpeaks, A-I and A-II. Both of these peaks are attributable to hybridization between O p-orbitals and the A-site cation, either Bi p-orbitals or La d-orbitals, in particular (for our doped cases), in the equatorial O atoms. These dominate the spectra when the experimental data are averaged over an entire unit cell^14^—as observed in Figure 5—because they have twice the abundance of the nonequatorial O atoms. As with the prepeak case, the d-density does not directly contribute to the simulated spectra since s→d transitions are electric-dipole-forbidden, but it instead acts by modifying the p-density of the O atom through hybridization.

It may be noted that the variation in the structure and intensity of the A-peaks is much greater in the doped systems than in pure bulk-like BFO. This is due to the introduction of the dopant atoms, which change both the chemical and physical environments of the O ions, thereby breaking the symmetry of the unit cell and making the atoms distinguishable by their different chemical environments. This is in addition to the longitudinal and equatorial environments, which are observed even within pure BFO.

In general, we observed that the magnitude of the A-peak depends on the proximity of the O atom in question to the La dopant. This can be seen in Figure 3. O2 is the nearest to La, whereas O8 is the furthest away. O4 has an O-La intermediate distance between that of O2-La and O8-La. This is unlikely to be due to the difference between equatorial and longitudinal sites around the elongated octahedra, since both O2 and O8 are in longitudinal sites, so the symmetry about the B-site does not appear to control the intensity of the A-peak.

A possible physical mechanism to explain this change in intensity is the difference in electronegativity between La and Bi. La is less electronegative than Bi (a Pauling electronegativity of 1.1 for La compared with that of 2.02 for Bi); this changes the energy of the unoccupied orbitals for the A-site cation, and therefore the energy of hybrid O-La orbitals occurs at a different energy to the O-Bi orbitals (as may be observed in the PDOS shown in Figure S3).

Now, by considering Fermi’s golden rule
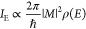
1where *I*_E_ is the intensity of a peak at energy, *E*, *M* is the transition matrix element, and ρ(*E*) is the density of unoccupied states at energy *E*; it can be seen that when some of the final states change their energy (due to the O being bound to a dopant rather than the original cation), some of the peak will change energy, leading to a drop in the intensity of the original, pristine-like peak.

The presence of La means that some density of final states is transferred from the energy range of the A-peak to the energy range of the B-peak. This effect is significant enough that the introduction of La dopants leads to a reduction in the A:B-peak ratio, with the presence of La reducing A:B from above 1 to below 1.

The change in intensity due to A-site doping may suggest a different interpretation from previous experiments (for example ref (23)), which have suggested that O vacancies (usually identified by the decrease in the integrated intensity of the O K-edge) are most readily found near the interface. While this study does not contradict this conclusion, it does indicate that a similar result can be obtained by the chemical doping of the A-site or even perhaps as a result of both doping and the presence of vacancies.

So far, it has been shown that the prepeak and A-peak regions of the spectra are highly sensitive to both A- and B-site dopings, with the prepeak intensity responding strongly to the amount of Mn present at the B-site of the system. While the A-peak’s features vary with the A-site doping, it is especially noticeable that the presence of La doping heavily suppresses the A-peak. Strain effects, however, result in a small chemical shift to the onset energy and otherwise only cause very subtle changes to the intensity of the spectra.

### 4.5 Simulated B-Peak

Figure 4 shows the B-peaks of O2, O4, O7, and O10 in the −0.20% strain case. O2 is in a longitudinal environment near La, whereas O7 is in the longitudinal environment but far from La; O4 is in the equatorial environment bonded to Mn, whereas O10 is in the equatorial environment, which does not contain Mn. These peaks are normalized against the same value as the peaks in Figure 3 and share common theoretical onset energies as they are from the same oxygen atoms. Peak B shows a splitting between two major subpeaks, referred to here as B-I and B-II. These two subpeaks are observable experimentally, and previous studies have shown that the B-I/B-II ratio can be used as a strong indicator to identify the phase of the BFO crystal under consideration.^61,64^

Both subpeaks in the spectra correspond to peaks in the Fe s-density (see the PDOS in Figure S3); however, the B-II peak is also at the center of an additional large region of Fe p-density that covers a range of 2 eV on each side of the central B-II peak. For the doped systems, there is a contribution from the Mn to these peaks as well, although the contributions from these dopants are shifted to a slightly higher energy due to the lower nuclear charge of Mn. The B-peak exhibits a similar grouping with respect to strain observed in the prepeak and A-peak. However, it is noticeable that for the equatorial O atoms (all O apart from O1, O2, O7, and O8), the B-peak shows a general trend of the B-I/B-II ratio increasing with Mn doping. Since two-thirds of the O ions in the simulation cells are in equatorial environments, this effect is also visible in all O averages shown in Figure S2.

The B-I peak is associated strongly with Fe orbitals, mainly the Fe s-orbital, although there is a significant p-orbital presence as well. There is no significant overlap from any Mn orbital present here, which suggests that the decreasing size of the B-I peak with increasing Mn doping is mainly due to the removal of Fe orbitals that may hybridize to serve as the final density of states for the excitation at this energy. The B-II peak is more complicated: while it centers around a second peak in the Fe s-density, it is dominated by Fe p-orbitals. Mn s-orbitals may also be found at this energy (around 12 eV above the edge onset), which may partially counteract any decrease in the B-II peak with the increasing presence of Mn in the system. This makes the B-I/B-II ratio a strong indicator of the presence of Mn. The Mn p-orbitals are found at 15 eV above the edge onset, as are the La p-orbitals. This leads to the presence of a shoulder to the right of the B-II peak.

This shoulder to the right of the B-II peak actually becomes a full peak for the low-strain cases of O2. This is not due to the contribution of the B-site cation but rather the presence of La at the A-site, which provides unoccupied p-density at this energy, increasing the feature from a shoulder to a definite peak.

The A-site also contributes significantly to the shape of the B-peaks, with La s-density present for all O atoms’ spectra apart from O7 and O8. The La s-density is located at the B-II peak. This is a relatively minor effect when compared to the Mn doping, as may be seen by comparing the spectra in Figure 4. The two spectra for atoms bonded to Mn (O2 and O4) have a significantly lower B-I peak than for the atoms that are not bound directly to Mn (O7 and O10). This occurs regardless of the Jahn–Teller-induced environment since O2 and O7 are in a longitudinal environment, whereas O4 and O10 are in equatorial environments.

By simulating changes to the peaks in spectra from structures of the type that may be expected to occur near a BFO/LSMO interface, we have built some degree of intuition for how the spectra should behave near an interface where a complex combination of strain and chemical effects will result in locally different chemical and crystalline environments. In the next section, we will illustrate how this knowledge can provide insights into a practical, experimental case.

### 4.6 Experimental BFO Spectra

It is clear from the simulation results that the precise form of individual ELNES peaks is heavily dependent on the local chemical environment of the individual atom where an energy loss event occurs. For complex systems such as the multiferroic interface of interest here, a high degree of variation of the crystal structure within a small area (e.g., due to strain or diffusion) is expected. This makes the choice of data processing and analysis of experimental data an important factor in aiding interpretation. We do neither expect nor aim to achieve a perfect match between the experimental results and the theoretical analysis: the use of highly doped supercells and the exclusion of channeling effects in the theoretical calculations, as well as experimental complexities arising from sample orientation (zone axis), preclude a quantitative match. The results below do, however, highlight some of the subtle variations in the spectra according to the local environment that were previously discussed.

Figure 5a shows a high-angle annular dark-field (HAADF) image of a BFO/LSMO sample acquired simultaneously with an EELS spectrum image, part of which is shown in panel (b) where the evolution of the O K-edge spectrum from BFO to LSMO is averaged horizontally parallel to the interface. This is displayed as a raster image using a false color intensity scale for clarity: individual atomic planes parallel to the interface are clearly observed (matching the corresponding HAADF contrast), while intense ELNES peaks appear as red features against blue troughs. Here, the change between the two-peak O K-edge structure of BFO^70^ (top) and the three-peak O K-edge structure of LSMO^71^ (bottom) can be seen clearly, with the interface region (between white dotted lines, as a guide to the eye) showing spectra with much broader, less intense peaks.

This flattening of the peaks has been previously reported in multiferroic thin films containing (i) interfacial cation intermixing^60,72^ and (ii) dislocations,^73^ yet a conclusion on the physical origin of this effect is still elusive. In both cases, it is generally accepted that this feature originates from the presence of oxygen vacancies.^23^ However, this is not a general consensus as several studies have also reported such flattening of the O K-edge in samples where no oxygen vacancies are suspected to exist^72^ and others have suggested interfacial cation intermixing as the possible origin for this feature.^74^ Probe channeling effects may also play a role in smoothing out the spectral features.

Here, we propose that the observed flattening may be rationalized, at least in part, by looking into the theoretical PDOS for the doped systems (see Section 4.2) and thus by the effect of sampling different chemical/structural environments. Examining the PDOS of the BFO and of LSMO (Figure S3), it is clear that the peaks of the La and Sr densities in the LSMO line up with the gap in density between the A- and B-peaks in the O K-edge of BFO. This suggests that an effective continuum of final states (and the associated flattening of the spectrum) may be sampled if several chemical substitutions with dopants are present locally through the sample thickness. Elemental maps of this sample (see Figure S5) indicate a degree of cation diffusion across the interface for both Fe (into the LSMO), and La and Mn (into the BFO). Elemental maps for Sr were not recorded. However, due to its lower abundance in the LSMO, less Sr may be expected to have diffused into the BFO. This qualitative argument offers one potential mechanism for the breaking of symmetry and the introduction of available states that could serve to fill the gaps between the BFO peaks and hence flatten the spectra.

Now, we turn our attention to the pristine BFO, away from the interface, to demonstrate that the trends revealed in our theoretical calculations regarding the contributions of individual chemical environments within a unit cell can be observed experimentally. As discussed previously, due to the Jahn–Teller distortion of the O octahedron, BFO contains two distinct local environments—one with four oxygen atoms per octahedron in an equatorial environment and another with two oxygen atoms in a longitudinal environment. These environments are represented schematically in Figure 2a. Several advanced statistical techniques exist to help remove noise and assist the analysis of the data.^75−77^ Here, we used a real-space template-matching averaging approach to obtain high signal-to-noise data while retaining spatially resolved information regarding the periodicity of the oxygen environments within the pure BFO structure. A pure BFO unit cell template, shown with a red box in Figure 5a, was matched using custom Python code across the spectrum image region of the BFO where no chemical interdiffusion was observed. The resulting average image, or patch, is shown in Figure 5a, with the corresponding O K map in Figure 5d, showing a good qualitative match to the schematic structure in Figure 5e, which once more illustrates the two distinct O environments—FeO, which corresponds to the longitudinal environments found in Section 4.1, and O_2_, which corresponds to the equatorial environment.

Figure 5f shows spectra extracted from this template-averaged data set. First, an average was made over the entire patch as shown in Figure 5c (black). This spectrum exhibits the typical features of the *R*-phase BFO discussed previously and is a good visual match to similar data in the literature.^64^ Assuming that the channeling effect may be qualitatively approximated using justifications based on the crystal structure, these experimental spectra would correspond to a weighted average over all chemical environments, as illustrated in Figure 1.

Additionally, the real-space averaging makes it possible to extract high signal-to-noise experimental spectra from different atomic columns and hence from different local crystalline and chemical environments (using a 2 × 2 pixel average centered on the FeO, Bi, and O_2_, as indicated in Figure 5f). These spatially resolved contributions all show the same broad structure of BFO. However, they also show subtle differences such as the suppression of the prepeak for the FeO (longitudinal) environment and a change in the onset of the B-I peak between FeO and O_2_ (equatorial) environments, all highlighted by arrows in Figure 5f.

The spatial variations in the experimental spectra as a function of probe position are known to be strongly affected by channeling of the probe through the crystal,^78^ thus requiring an additional numerical framework expressed in terms of a weighted dynamical form factor,^27^ with the net effect that significant mixing of environments should be expected away from atomic columns (e.g., the O signal obtained when the probe is located on top of the “Bi” column). Nevertheless, the effects observed experimentally broadly follow the trends established numerically for the distinct O environments, with varying relative strengths of the prepeak (P) and the appearance of additional intensity on the high-energy-loss shoulder of the A-II peak. This suggests that model calculations as carried out here still provide useful insights into the local environment contribution to spatially resolved spectroscopy data.

The general principle that the simultaneous sampling of multiple environments could be partly responsible for the observed flattening of fine structure may also be extended to the O K-edge at different positions around a dislocation core (see Figure S5). For this defect type, there is not expected to be significant chemical doping (within the core itself), but nevertheless the wider symmetry of the material may be disrupted,^79^ leading to the generation of multiple local chemical environments.

It is worth noting that any change that makes one atom distinguishable from another will yield a slight change in its spectrum, and generally speaking, the more significant the change that breaks the symmetry, the greater the variation between the spectra. Due to the “short-sightedness of nature”,^80^ these perturbations have their largest effect close to where the symmetry is broken and do not provide uniform shifts.

We emphasize that this principle says nothing about the origin of the broken symmetry, which may be attributable to a wide range of factors, including strain gradients, dopants, point defects, and O vacancies (which have often been discussed in the context of dislocations^79^) but also to any other mechanism that reduces the symmetry.

These observations clearly indicate that there is great value in investigating the effect that the multiple localized chemical environments can have on the O K-edge spectral features.

### 4.7 Relative Magnitude of Effects

In addition to the material-specific changes we have highlighted in this work, it is also possible to draw out some more general inferences about the size of effects that certain features might be expected to have on an oxygen K-edge within an oxide perovskite. The general principle that underlies this is that the larger the perturbation to the electronic structure, the larger the effect on the EELS spectrum, as one would reasonably expect.

The largest effect that will be commonly seen when considering theoretical results is the core hole effect when the unoccupied state has a significant contribution from the atom where the excitation originates.^51^ When the final state has a lower contribution from the original atom, this effect may be less significant, however. The core hole effect is also unlikely to be of significant concern when interpreting experimental spectra; the magnitude of change required to affect whether a certain atom contributes to the unoccupied states or not would be on the order of changing which material is being probed.

The next largest effect is the change of chemical identity of an atom—whether through deliberate doping or interdiffusion between layers. For nonisovalent substitutions, this can change which states are occupied or not, which could have a significant effect on the structure of the spectrum near the edge onset, especially when the dopant atoms are present in high enough concentration that they do not merely express themselves as trap states.

In addition to this, the unperturbed (*in vacuo*) energy levels of any individual atom will be different from that of an atom of any other species. By introducing a new element, the energy levels—and accordingly the peak energies—will have their positions altered, giving a large qualitative change to the spectrum.

Next, structural changes that affect the symmetry of the system (such as phase transitions or rearrangement of atoms around defects and vacancies) are likely to play a significant role. In this case, there will be more complex energy distributions of bands, thereby making many more transitions viable. Any large-scale breaking of symmetry is likely to cause significant smearing to the spectrum, as the number of nondegenerate energy levels increase.

Finally, perturbations that lower but do not totally break the symmetry of the system such as small strains (lower than required to induce a phase transition or defects) will cause small perturbations to the spectra, generally involving subtle changes to the relative intensity of peaks and peak positions. Excepting the case of a very high purity crystal, the authors do not believe that these small strains should be distinguishable within an experimental spectrum.

## 5 Conclusions

This work presents a simplified illustration of some of the physical mechanisms underlying changes experimentally observed in the spectral features of the O K-edge of complex systems such as multiferroic thin films through the joint use of experimental and computational techniques. By comparing the spectral features from small regions and the structure of the different local chemical environments that produce them, it has been possible to draw conclusions about the main factors that influence the overall spectra for doped and strained perovskite systems, such as within BFO/LSMO thin films.

Individual contributions to the EELS spectra were investigated computationally for the oxygen atoms within two cases of doped BFO/LSMO systems. It was found that while the overall two-peak structure of BFO was maintained, the introduction of dopants broke the local symmetry, creating several distinguishable local environments. This gives rise to significant changes in the spectra. It was also observed that strain alone does not significantly affect the features of the O K-edge spectra, instead only applying a small chemical shift to the onset energies. This suggests that the BFO/LSMO interfacial structure is relatively resistant to the small strains introduced by lattice-matching between the electrodes or substrate, but that greater changes in the spectra may emerge from other growth-related effects such as structural defects and chemical diffusion.

Through examining the experimental O K-edge ELNES from a BFO/LSMO interface and from a region inside the BFO thin film, we show that even simple, model calculations help to rationalize how the local O environments contribute differently to the overall spectra. In particular, this allows us to interpret the well-reported flattening of the O K-edge signal at the interface as arising at least, in part, from the sampling of O atoms in multiple different local chemical environments induced by the presence of multiple cations near the interface.

This work emphasizes the importance of keeping the local variations within the sample and spatial averaging in mind when analyzing spectra from complex systems, as the combination of multiple local environments induces significant effects on the interpretation of the experimentally acquired spectra.

## Supporting Information Available

Further site-resolved spectra, projected densities of states, and elemental mapping for experimental samples (PDF)
