# Supplementary material for: Effects of Multiple Local Environments on Electron Energy Loss Spectra of Epitaxial Perovskite Interfaces
Source: J Phys Chem C Nanomater Interfaces. 2022 Dec 8;126(50):21453–66. doi: 10.1021/acs.jpcc.2c06879 (PMC9791663; doi:10.1021/acs.jpcc.2c06879)
Supplement: Supplementary file 1 — jp2c06879_si_001.pdf [file jp2c06879_si_001.pdf]

The Effect of Multiple Local Environments on Electron Energy Loss Spectra of Epitaxial Perovskite Interfaces

Robert A. Lawrence [a,*], Quentin M. Ramasse [b,c], Kristina M. Holsgrove [d], Daniel Sando [e], Claudio Cazorla [f], Nagarajan Valanoor [g], and Miryam A. Arredondo [d,*]

a Department of Physics, University of York, Heslington, North Yorkshire, YO10 5DD, United Kingdom. *E-mail: robert.lawrence@york.ac.uk
b SuperSTEM Laboratory, SciTech Daresbury Campus, Daresbury, WA4 4AD, United Kingdom
c School of Chemical and Process Engineering and School of Physics and Astronomy, University of Leeds, Leeds, LS2 9JT, United Kingdom.
d School of Mathematics and Physics, Queen's University Belfast, Belfast, BT7 1NN, Northern Ireland, United Kingdom. *E-mail: miryam.arredondo@qub.ac.uk
e School of Physical and Chemical Sciences, University of Canterbury, ChristChurch 8140, New Zealand
f Departament de Fisica, Universitat Politecnica de Catalunya, Barcelona E-08034, Catalonia, Spain.
g School of Materials Science and Engineering, University of New South Wales, Sydney, NSW 2052, Australia

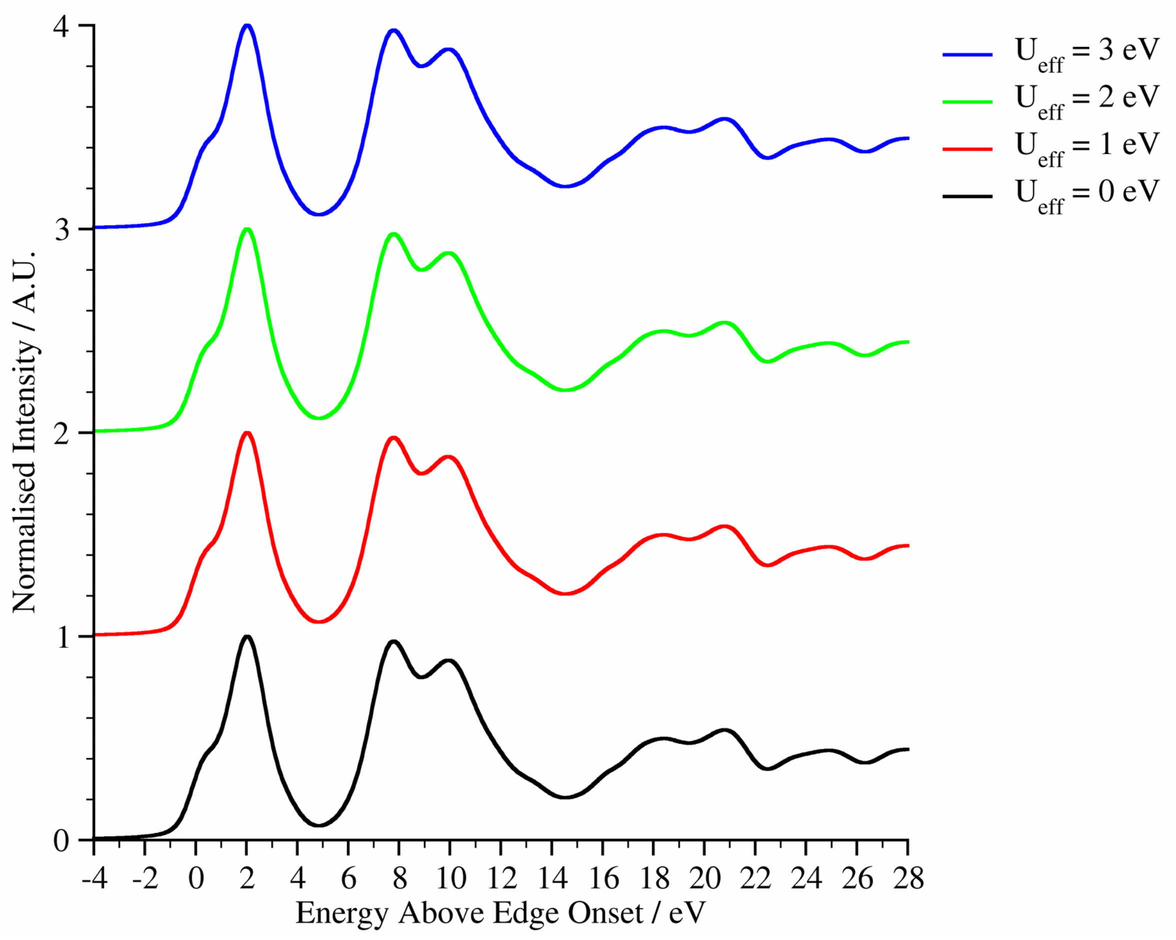

Figure S1. The effect of varying Hubbard-U on the spectra. The effective Hubbard U parameter was varied for BFO whilst the geometry was kept fixed. Due to the dipole correction, the d-density is not directly included in the spectra since s to d transitions are dipole forbidden. There are no

observable changes to the spectra due to this variation of Hubbard-U, suggesting that any effect it has on the spectra is mediated through the effect it has on the overall geometry of the system.

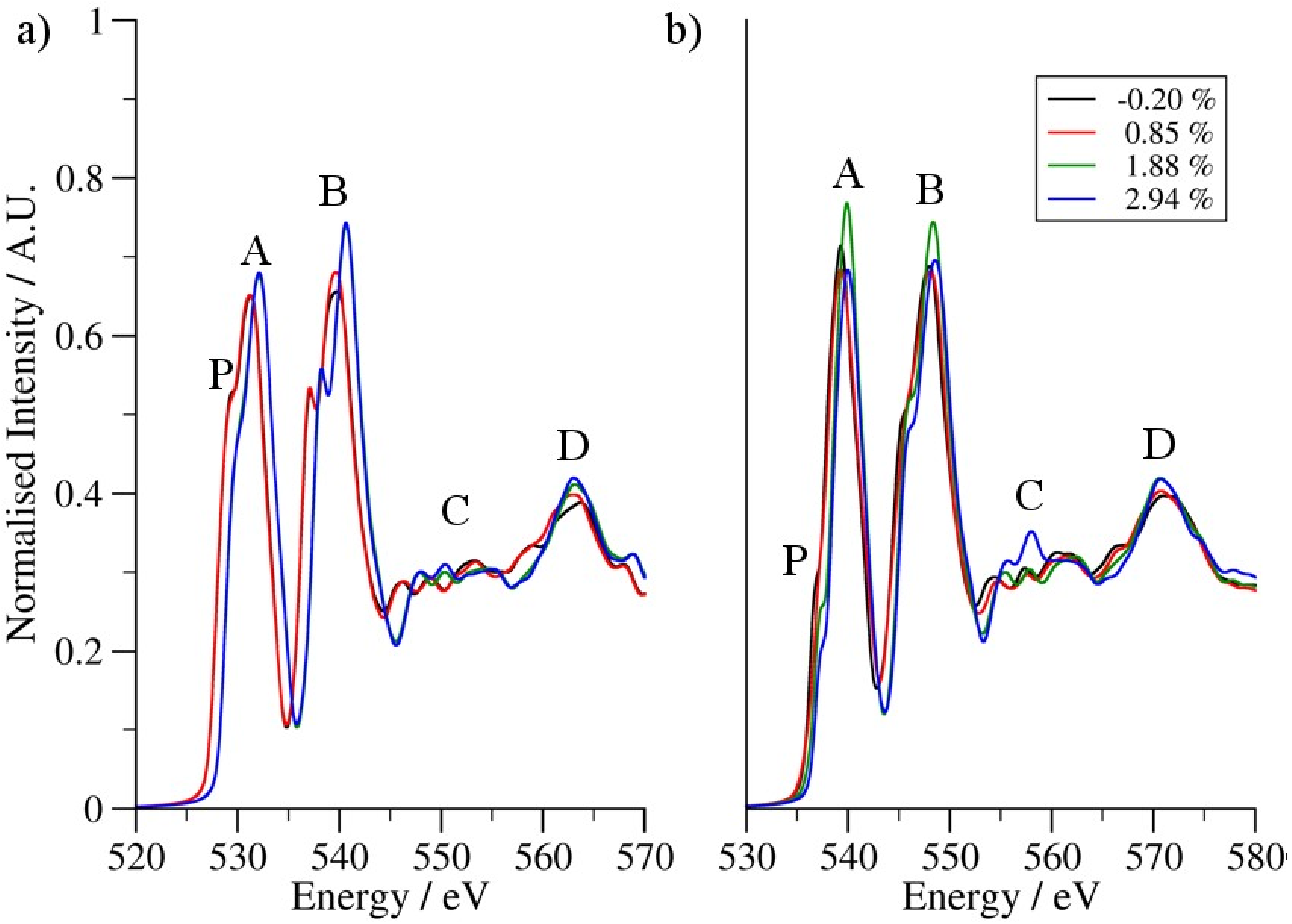

Figure S2. Average (whole unit cell) spectra for the different strain cases used in this work. Panel a) shows the 25% Mn and 25% La doped system, whereas panel b) shows the 50% Mn and 25% La doped system. Note the clear difference in pre-peak intensity and the change of the B-I peak from a distinct peak to a shoulder.

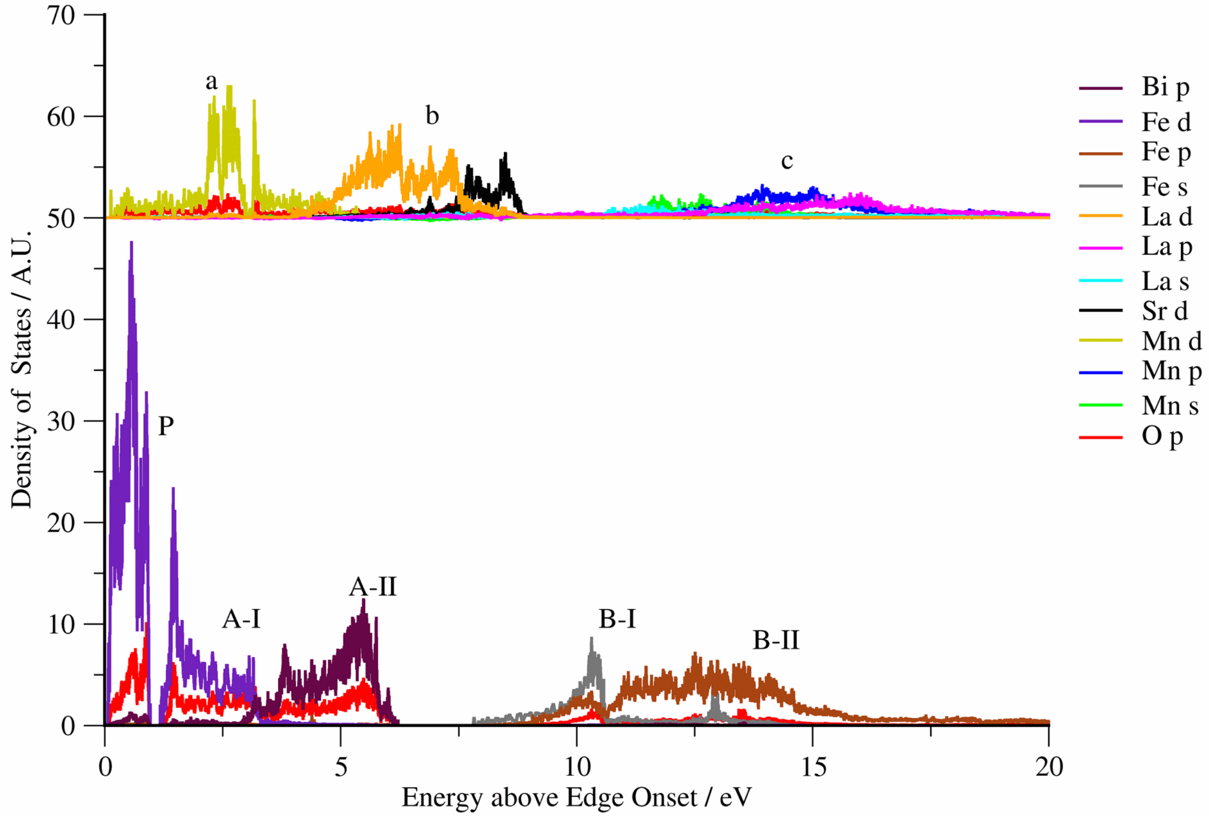

Figure S3: PDOS for pure BFO (below) and LSMO (above). Note that the b-peak of LSMO aligns with the gap between the A-II and B-I peaks of BFO. Due to PDOS not including selection rules, the d-density makes a much more significant contribution to the PDOS than to the spectra.

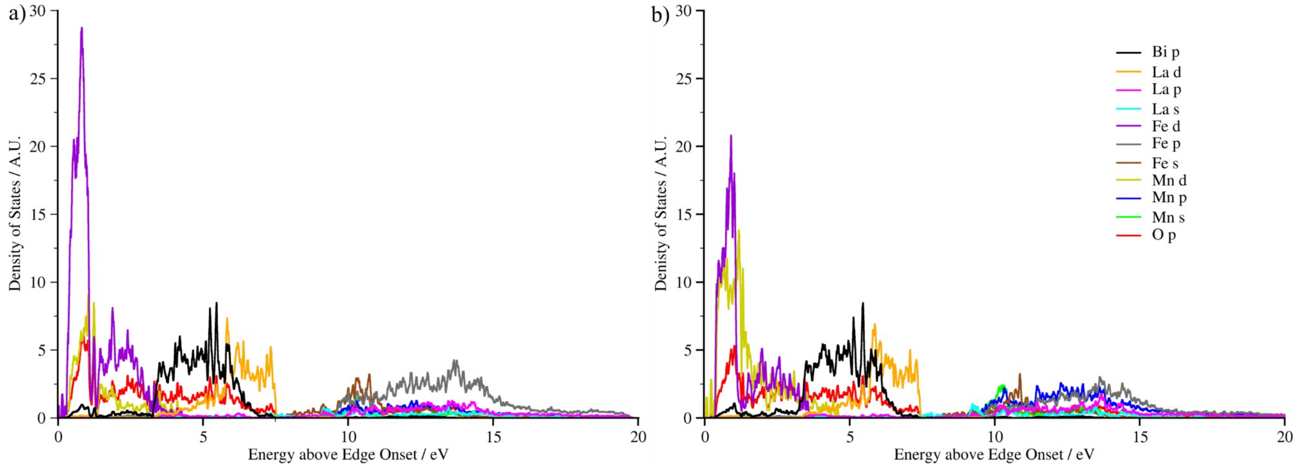

Figure S4: PDOS for the doped BFO. Both cases contain 25% La doping. The panel a) contains 25% Mn, whereas panel b) contains 50% Mn. Note that the La contributes density in the region of the B-peak, and that the structure of the Mn d-density changes with dopant concentration.

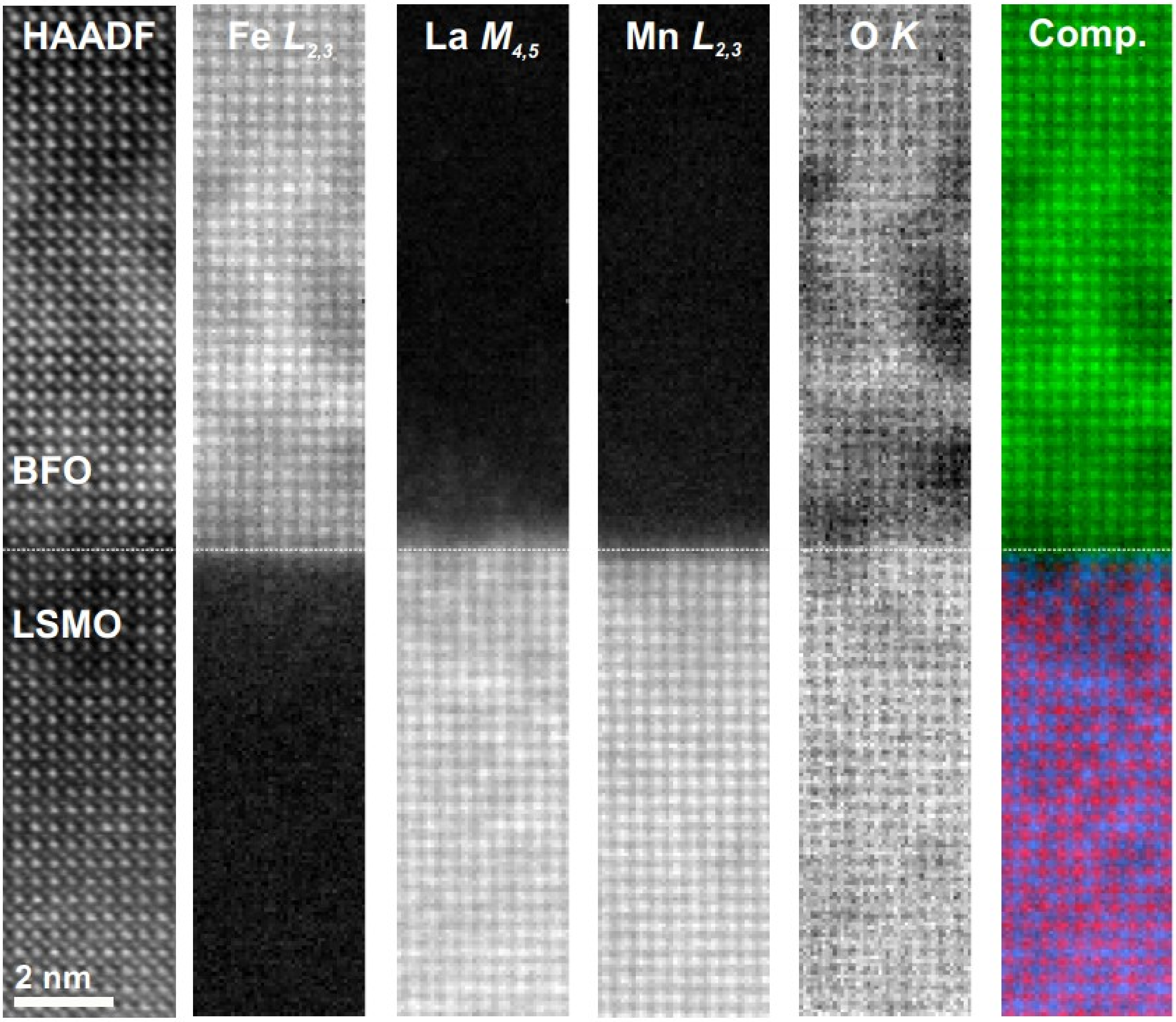

Figure S5. Elemental maps indicating diffusion for the BFO/LSMO interface. a) shows the HAADF for the mapped region. Note the considerable diffusion of Fe and La across the interface, and the slight diffusion of Mn. The Sr edge was not recorded, however it may be expected to diffuse into the BFO at a similar rate to La, although with less total Sr diffusion due to its lower concentration in the LSMO.

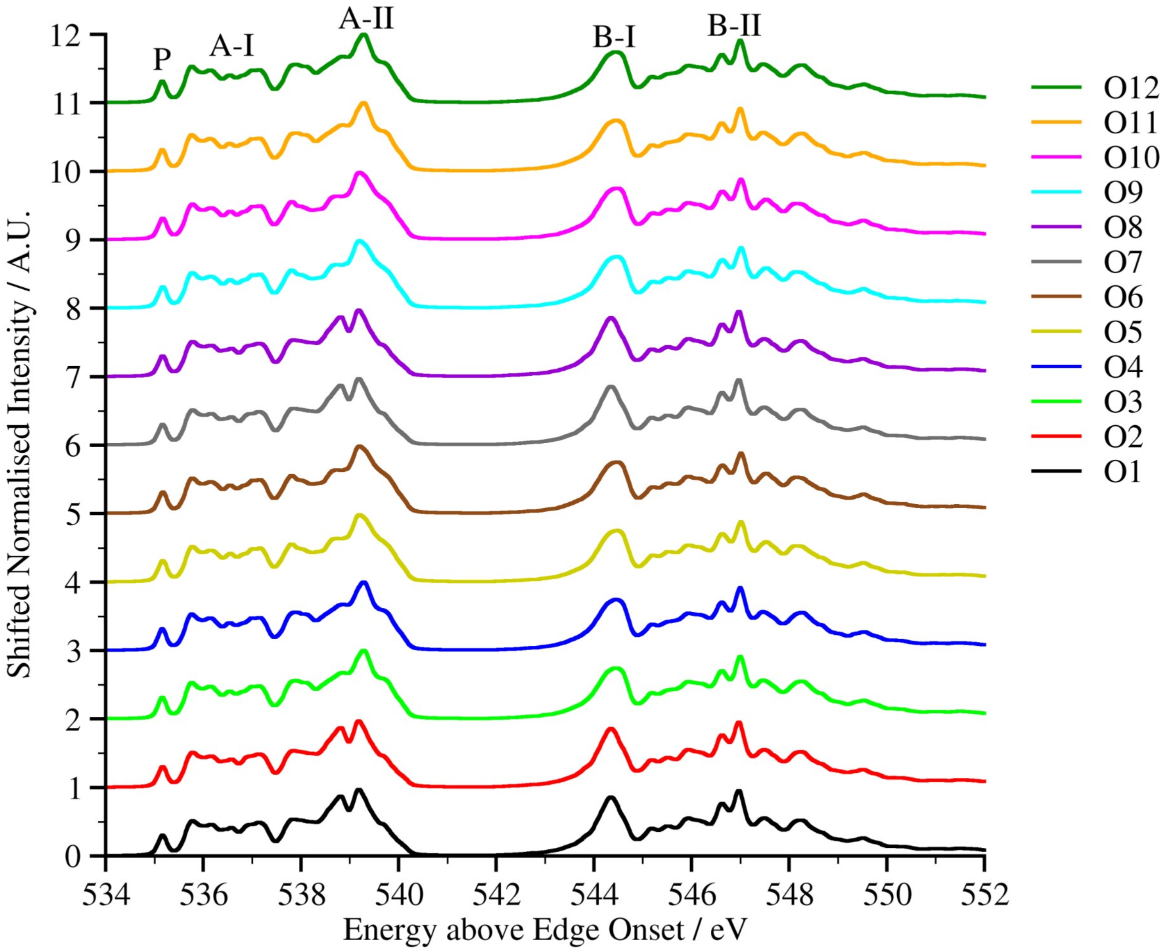

Figure S6: Undoped BFO spectra. Spectra 1, 2, 7, and 8 come from a longitudinal environment, whereas spectra 3, 4, 5, 6, 9, 10, 11, and 12 come from an equatorial environment. The changes to the spectra between these environments consist of a changing shape of the B-I peak and a splitting of the A-II peak into two distinct sub-peaks.

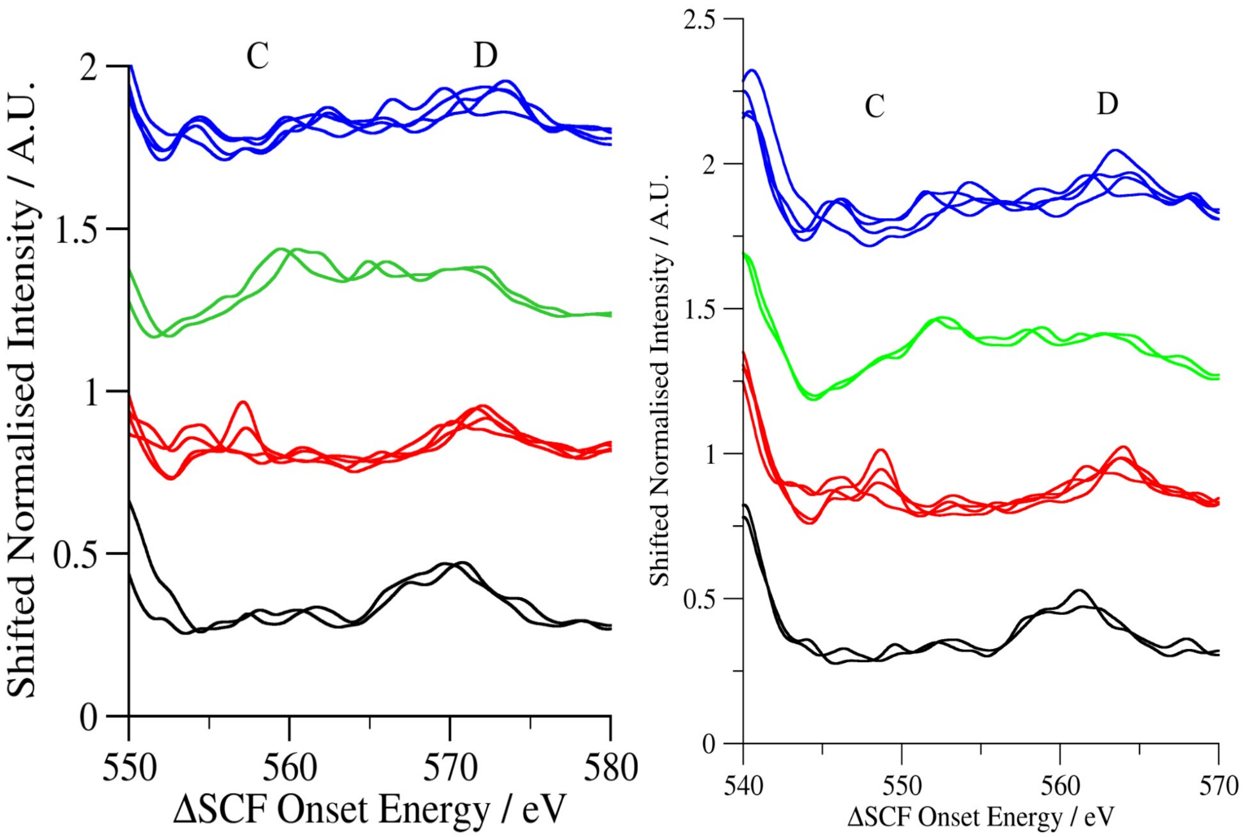

Figure S7. C&D peaks (50% Mn left, 25% Mn right). Spectra are grouped by environment. Blue is the equatorial (not near Mn) environment, green is the longitudinal (not near La) environment, red is the equatorial (near Mn) environment, and black is the longitudinal (near La) environment. Note these spectra are very strongly grouped by local environment. The difference in apparent onset energy is due to the systematic variation in theoretical onset energy between the two doping cases, which is not expected to be a genuine physical effect.

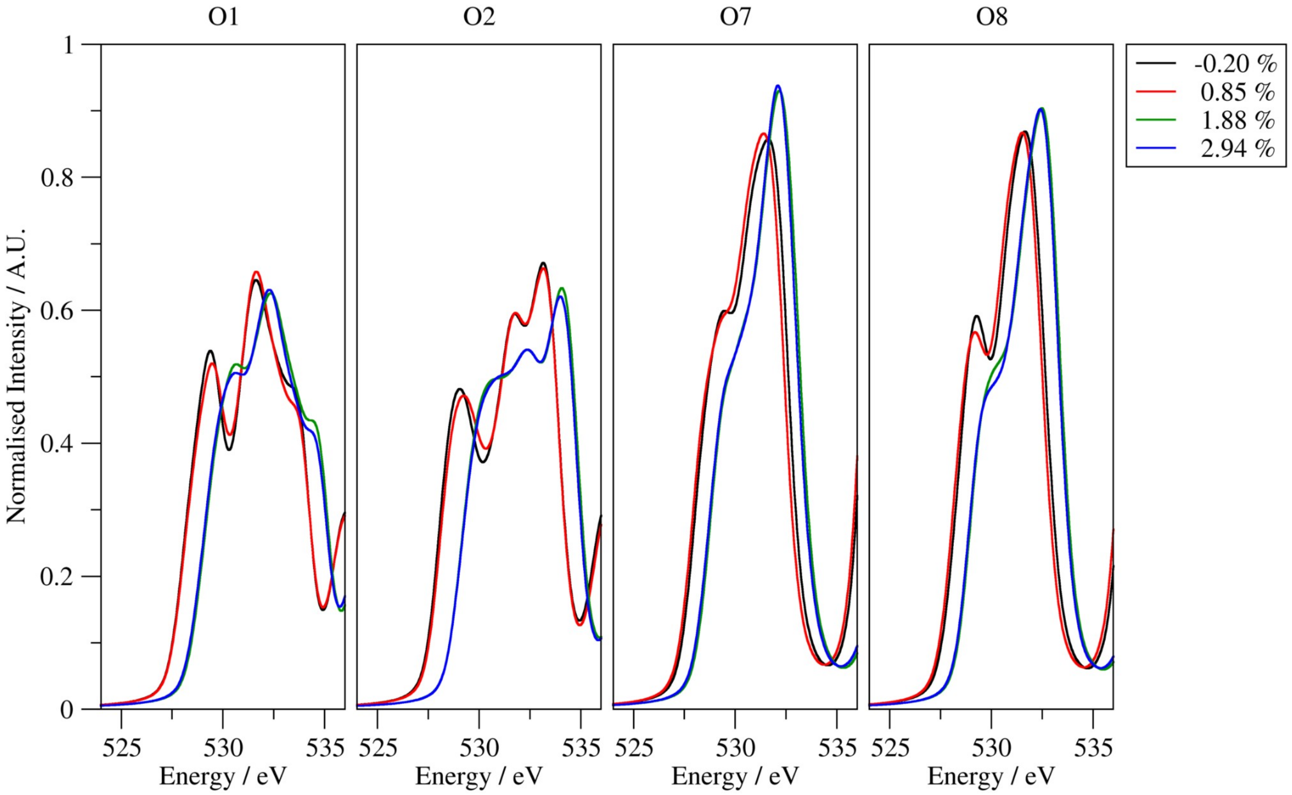

Figure S8. Detailed view of P and A peaks for atoms in the longitudinal environment for the 25% Mn doped system over 4 strain values. Note that the jump in out-of-plane lattice parameter causes a shift to the onset. All of the following supplementary figures will show the variation of various peaks over strain for the several local chemical environments found in our computational BFO model.

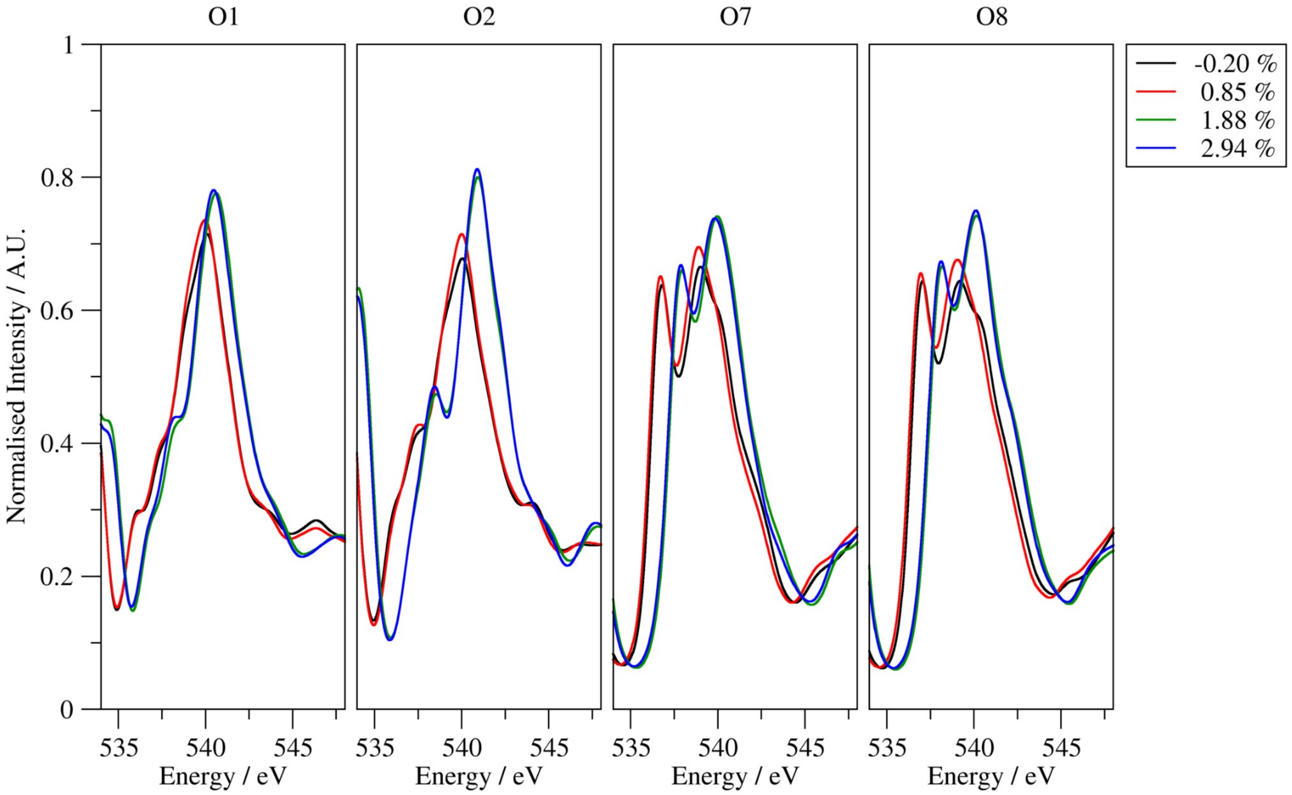

Figure S9. Detailed view of B-peaks for atoms in the longitudinal environment for the 25% Mn doped system over 4 strain values. Note O1 and O2 occur near La, whereas O7 and O8 do not, which has a significant impact on the qualitative shape of the peak.

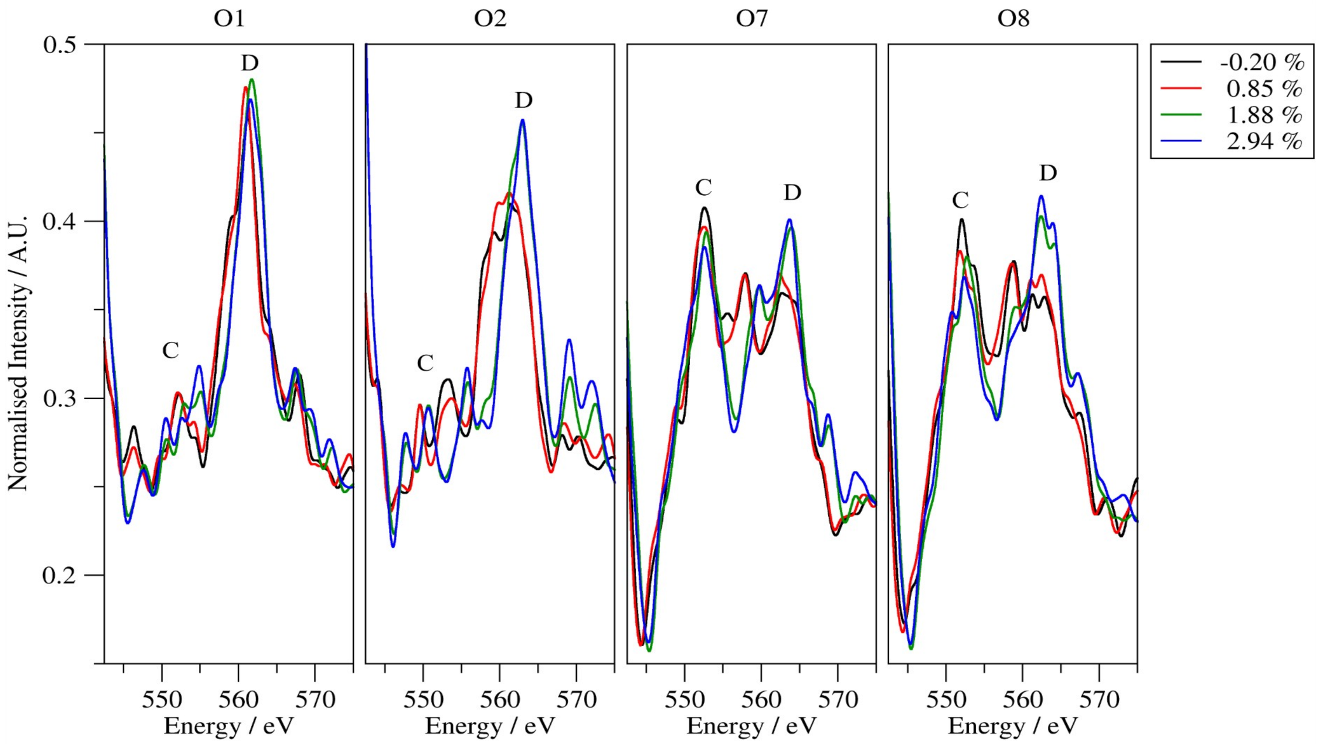

Figure S10. Theoretical C & D peaks for O atoms in the longitudinal environment of the 25% Mn system. Note that the jump in onset which was marked for the A and B peaks of these atoms is no longer present – perhaps suggesting that these peaks are strictly EXELFS rather than ELNES.

Nevertheless they remain highly sensitive to the local chemical environment – with the result that they are hard to distinguish experimentally.

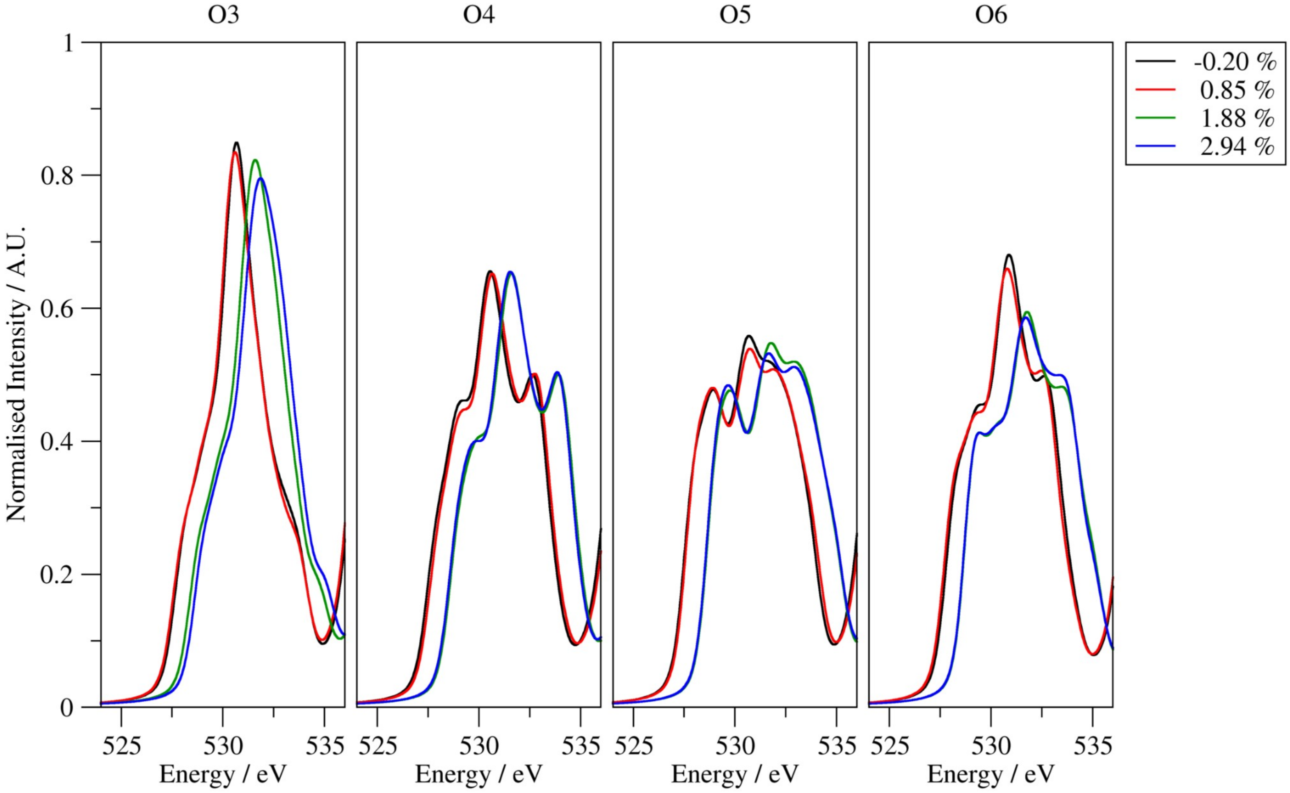

Figure S11 Pre and A peaks for the equatorial near Mn environment for the 25% Mn case.

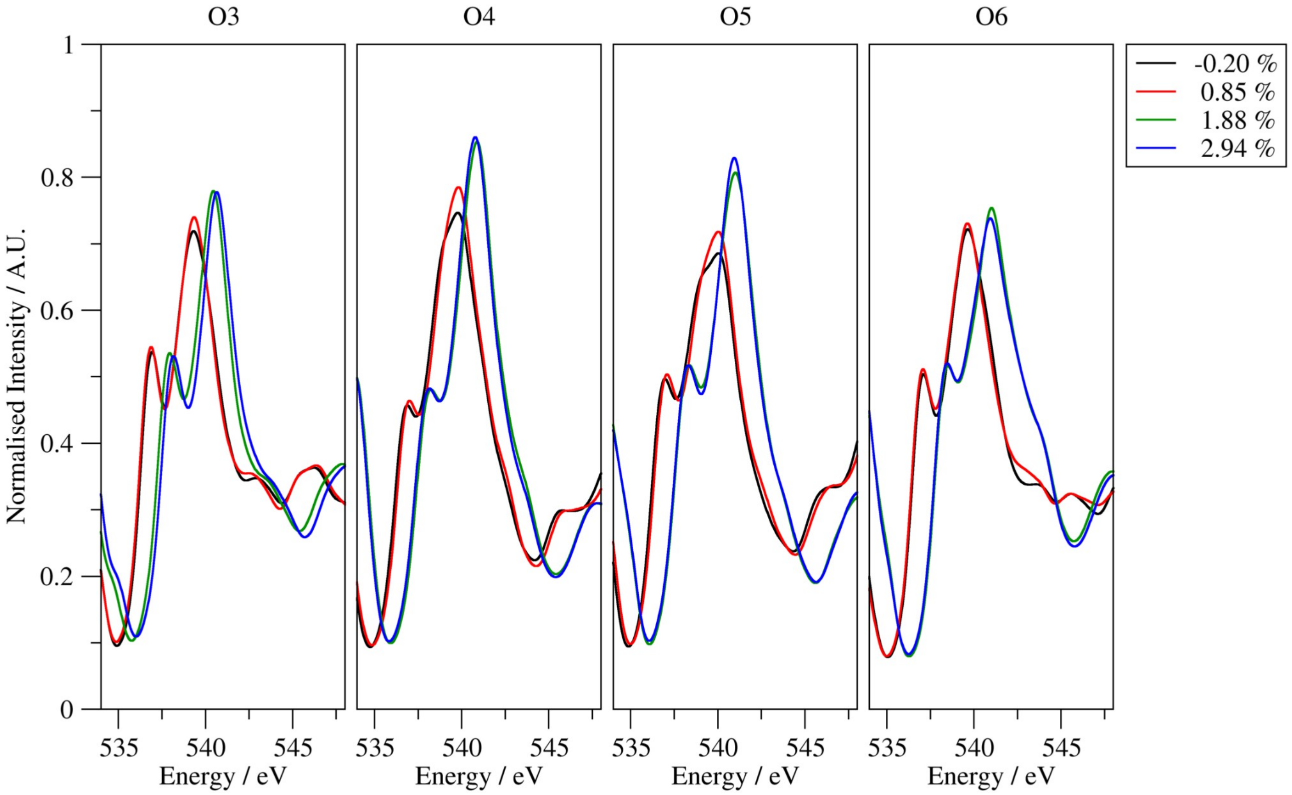

Figure S12. B peaks for the equatorial near Mn environment for the 25% Mn case. The subtle variations in the peak structures between atoms are likely to be to do with the slight variations in the bond angles due to the octahedral tilts.

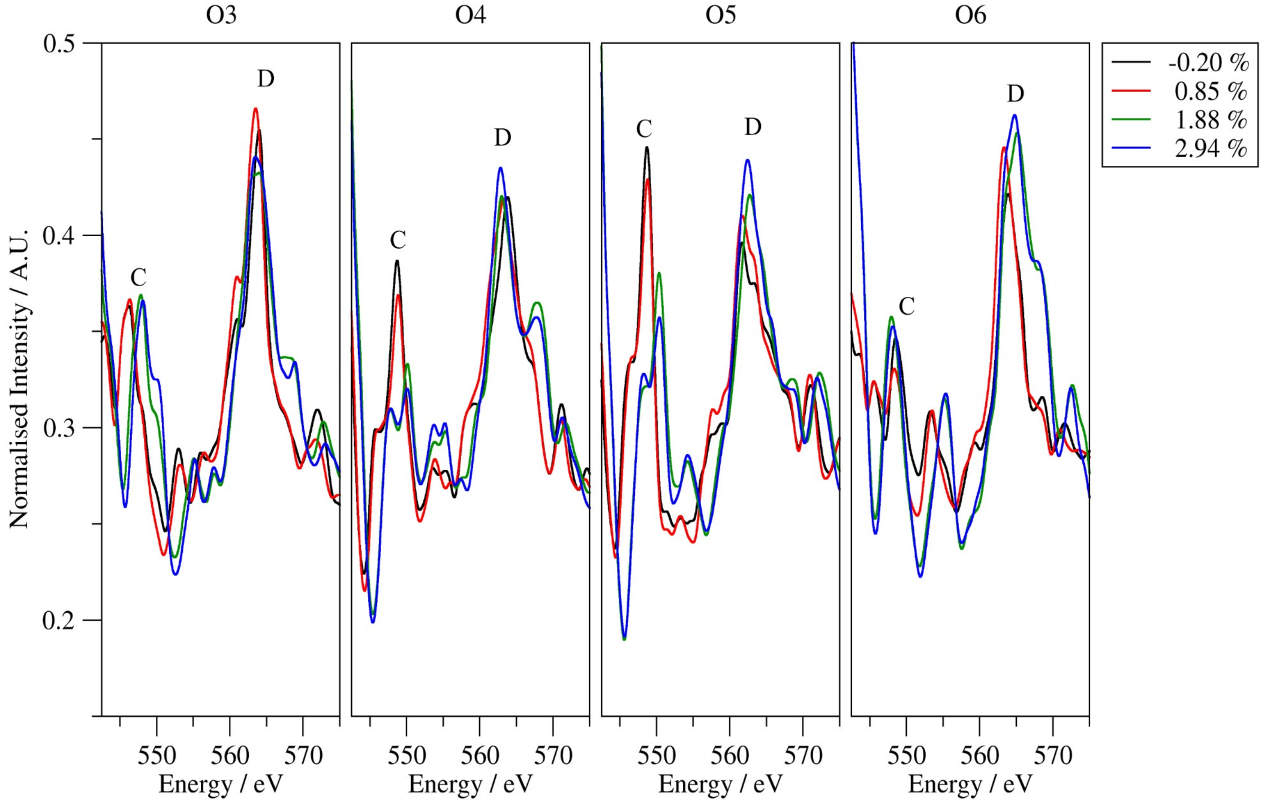

Figure S13. C and D peaks for the equatorial near Mn environment for the 25% Mn case. Note the difference is structures between these peaks and those for the other environments presented in this

S.I. Whilst in principle these peaks could be used to distinguish the local environment, in practice channelling and sampling effects make it very unlikely that these peaks could be separated.

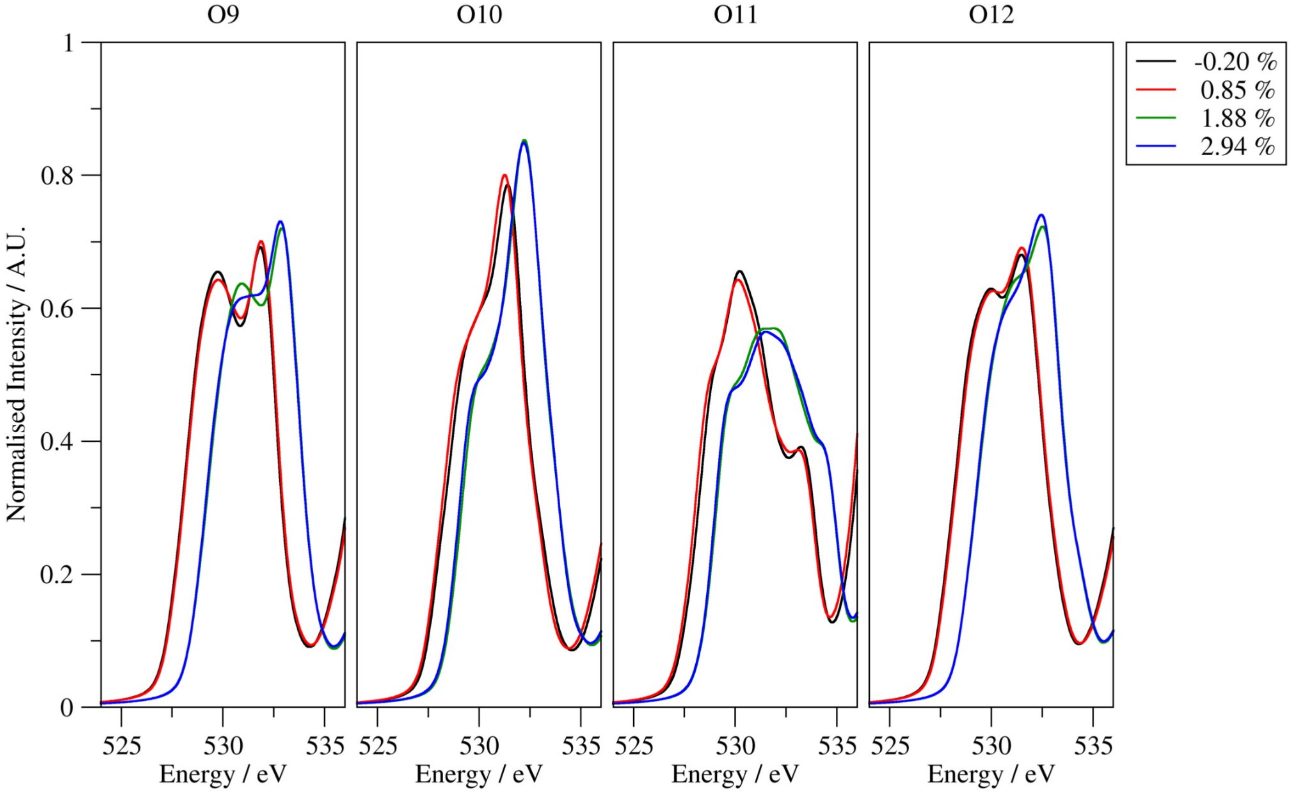

Figure S14. Pre and A peaks for the equatorial near Fe (not-near Mn) environment for the 25% Mn case.

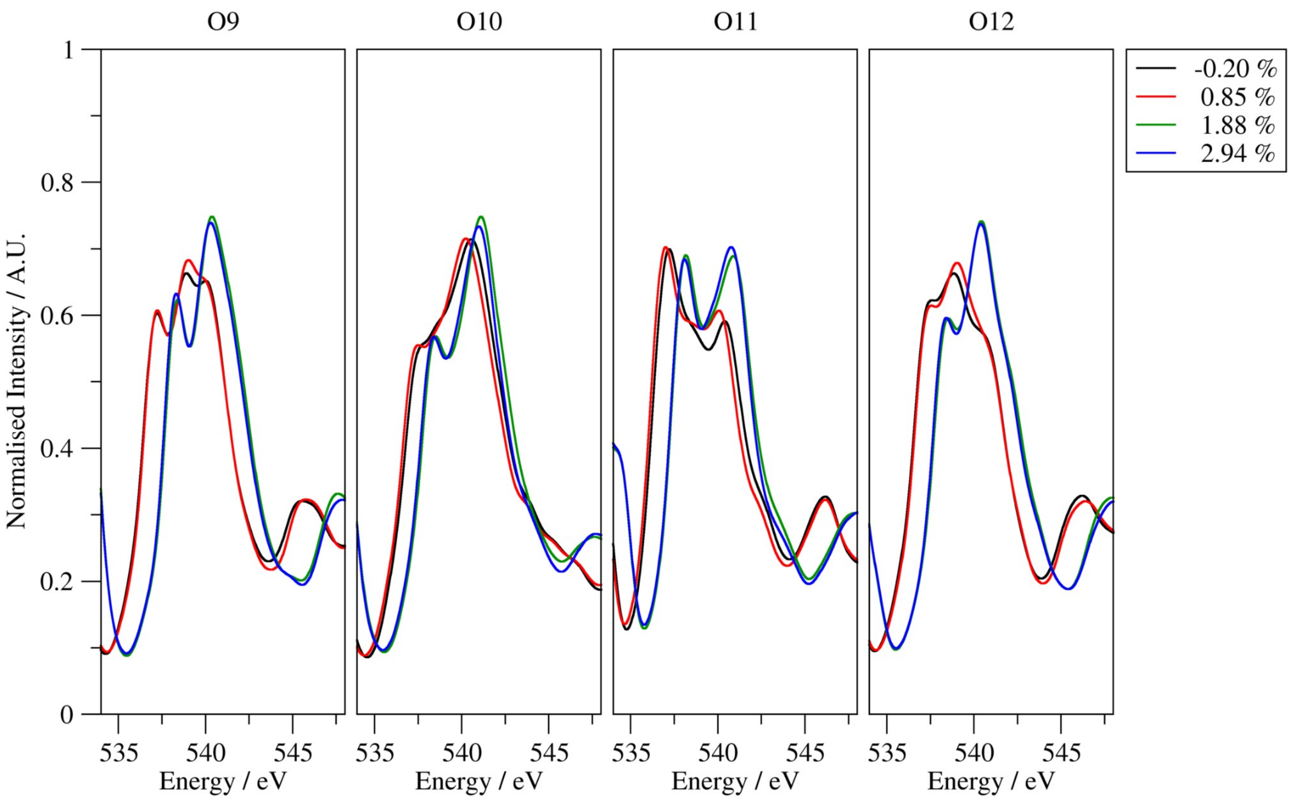

Figure S15. B peaks for the equatorial near Fe (not-near Mn) environment for the 25% Mn case. Note the much more even B-I:B-II ratio here compared to the equatorial near-Mn case (sup.Fig.13)

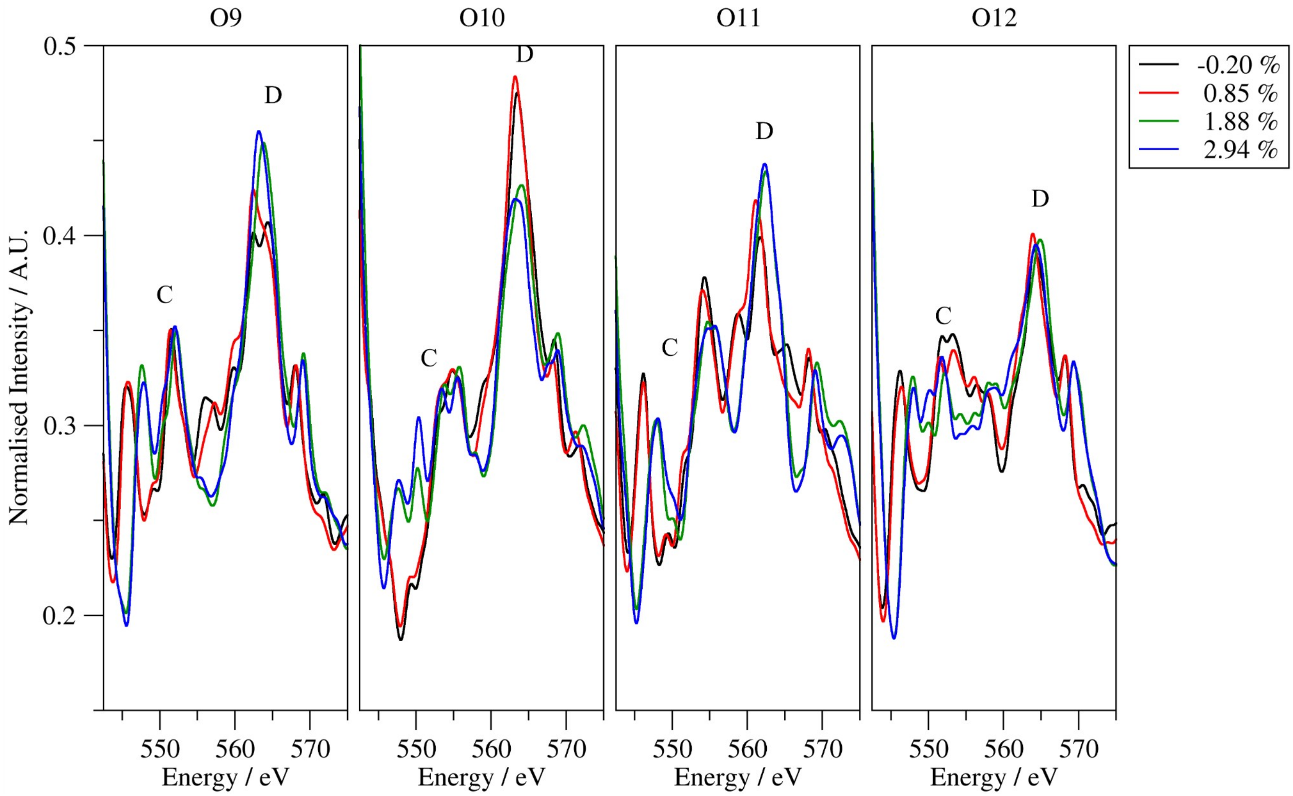

Figure S16. C and D peaks for the equatorial near Fe (not near Mn) environment for the 25% Mn case.

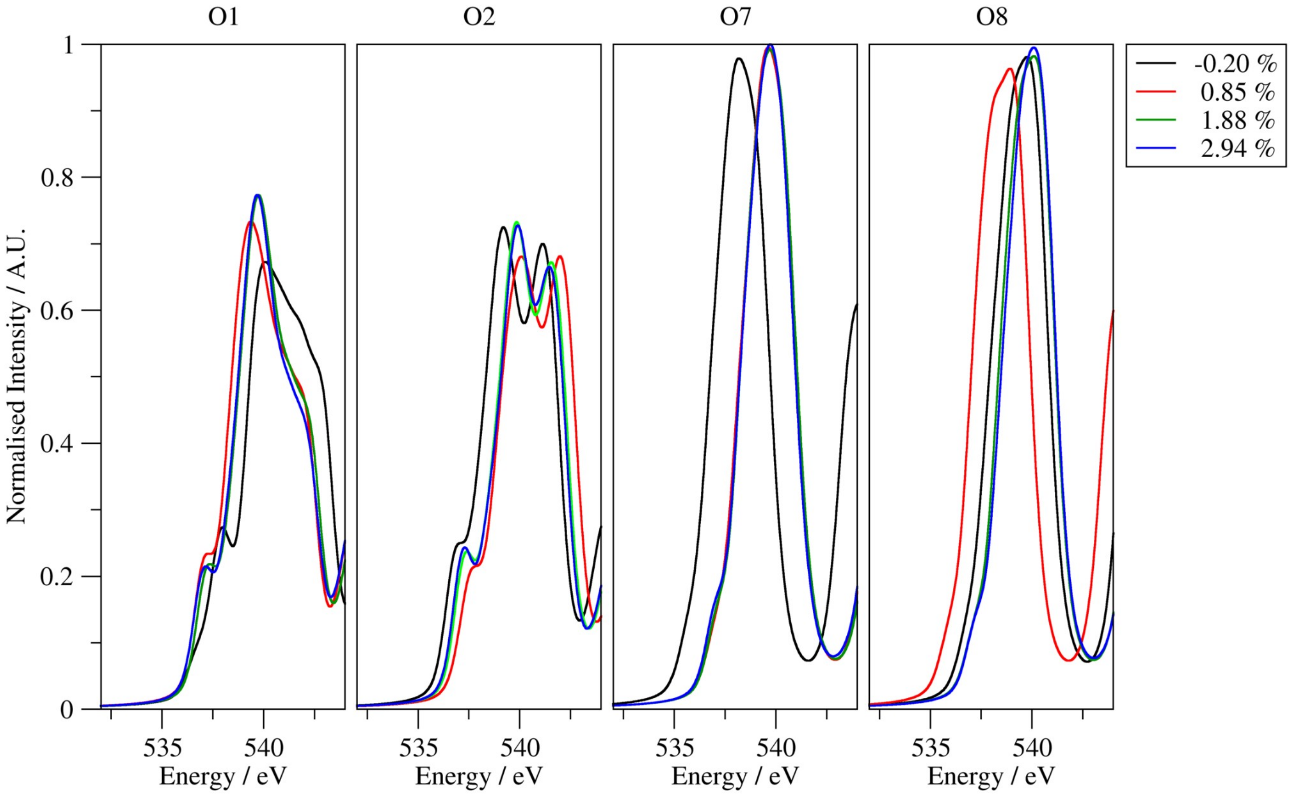

Figure S17 Pre and A peaks for the longitudinal atoms in the 50% Mn doping case. Note the strong distinction between O1/2 and O7/8 caused by the proximity of the La atom to O1 and O2.

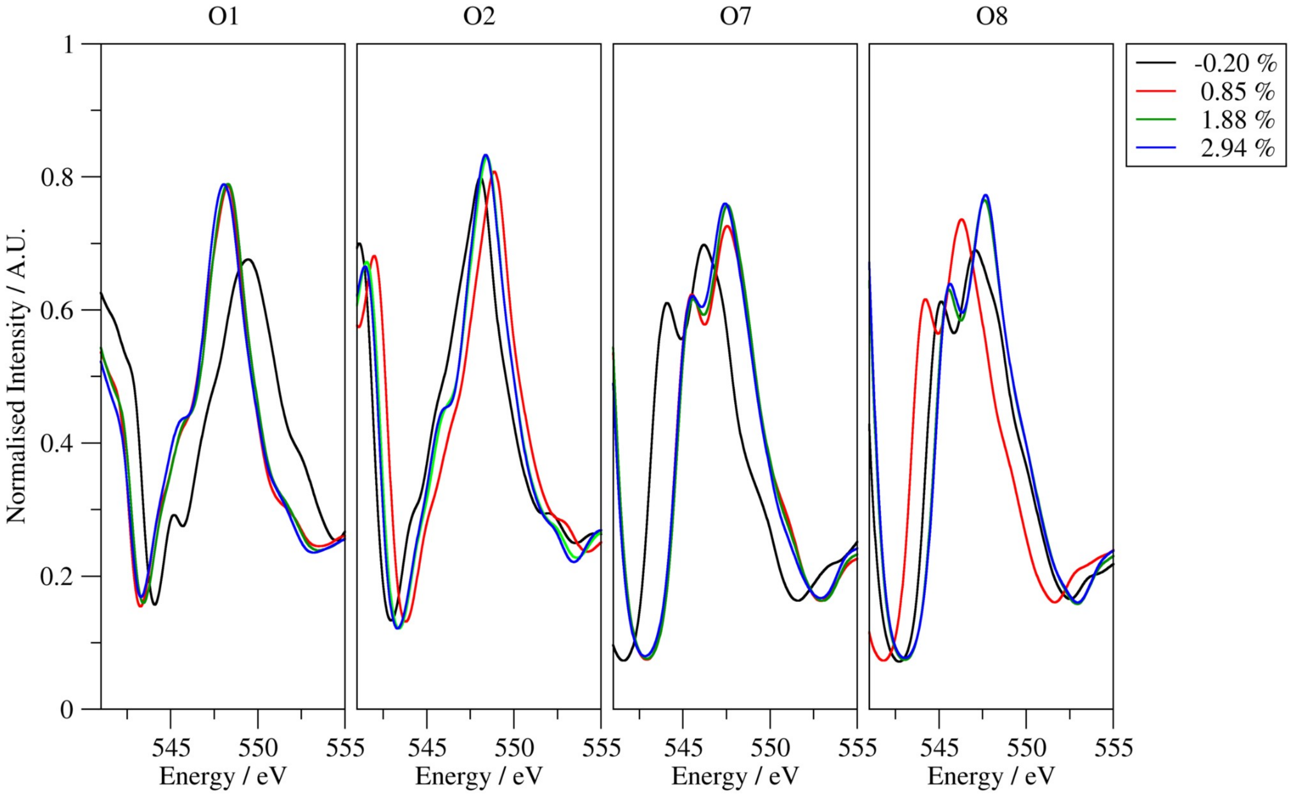

Figure S18 B peaks for the longitudinal atoms in the 50% Mn doping case.

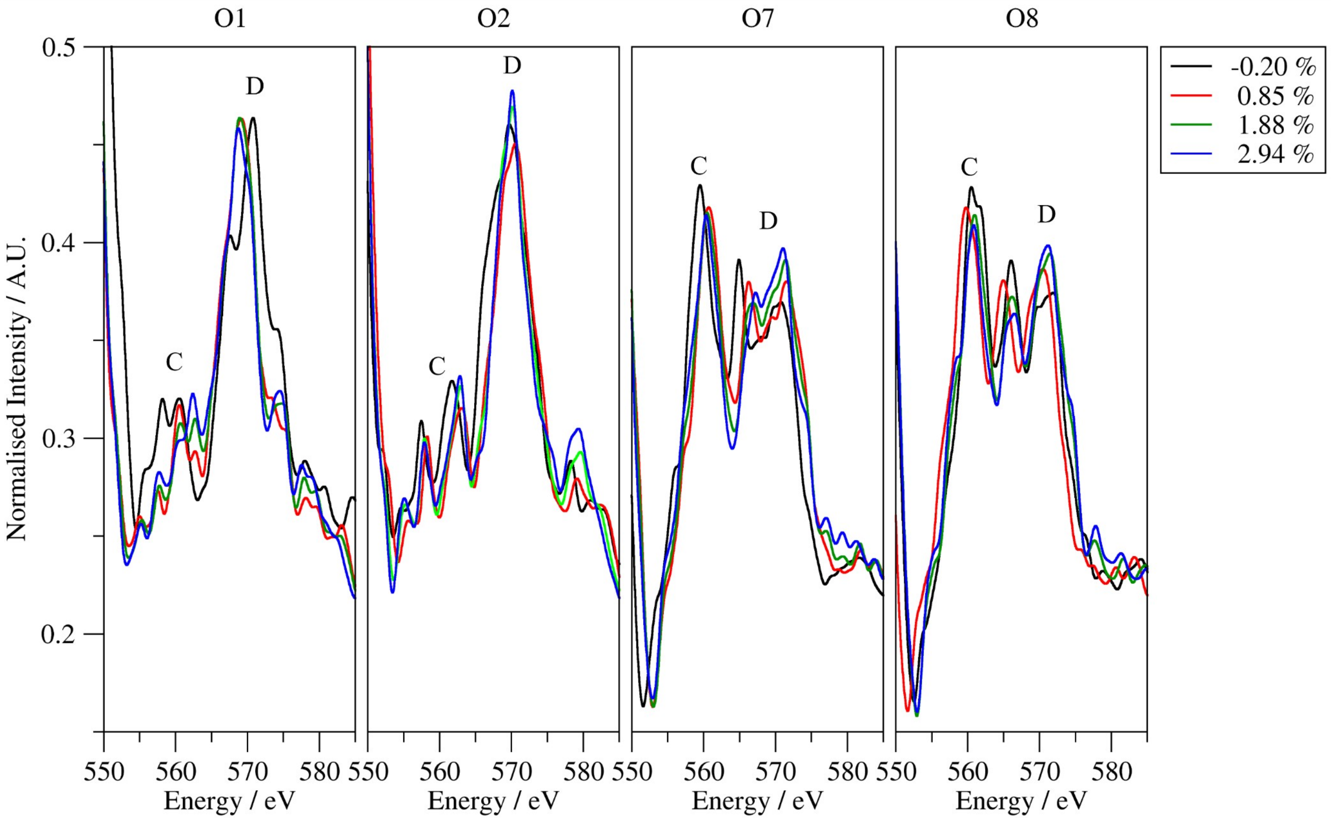

Figure S19. C and D peaks for the longitudinal atoms in the 50% Mn case. Note the strong change in peak structures depending on the proximity of the La atom.

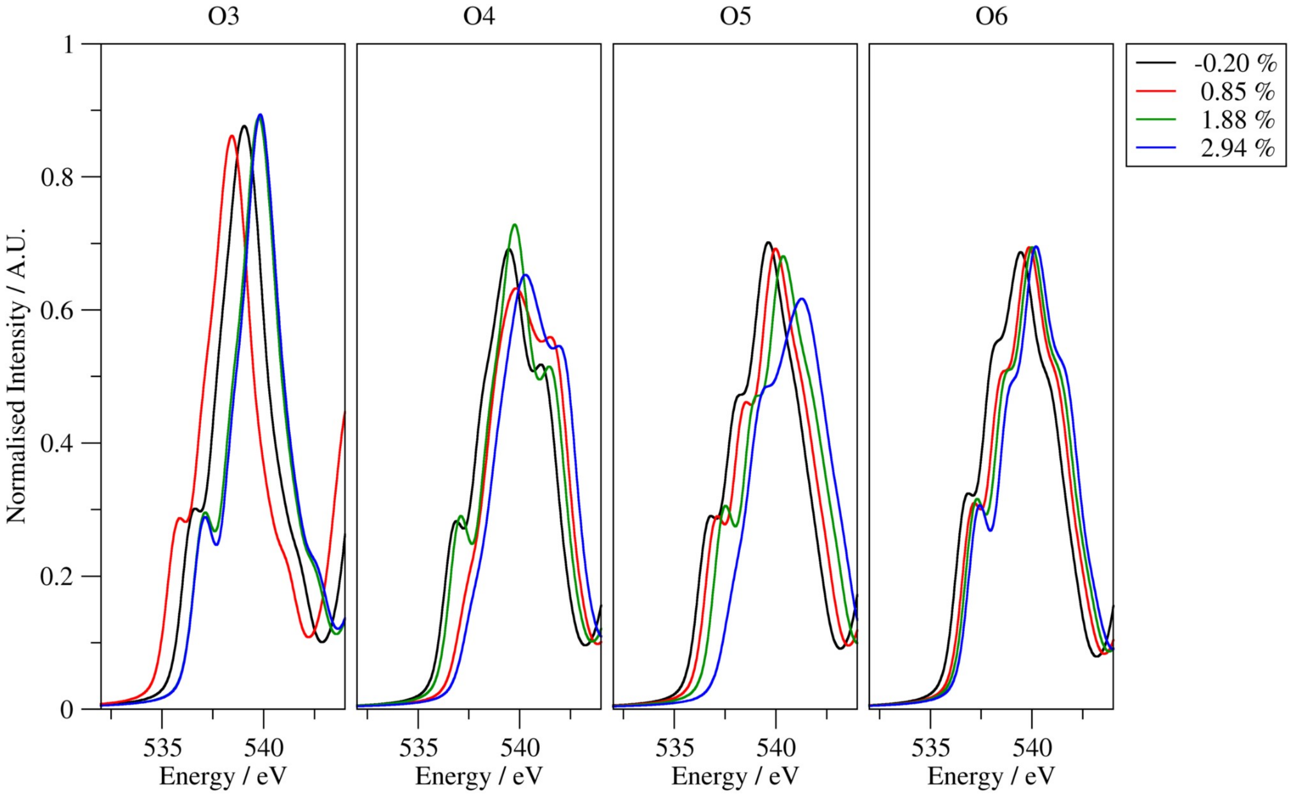

Figure S20. P and A peaks for the equatorial near Mn case for the 50% Mn doped system. Note that the jump of lattice parameter with strain found for the 25% Mn case did not occur here; rather the lattice parameter varied smoothly – accordingly no jump in the onset energy was found.

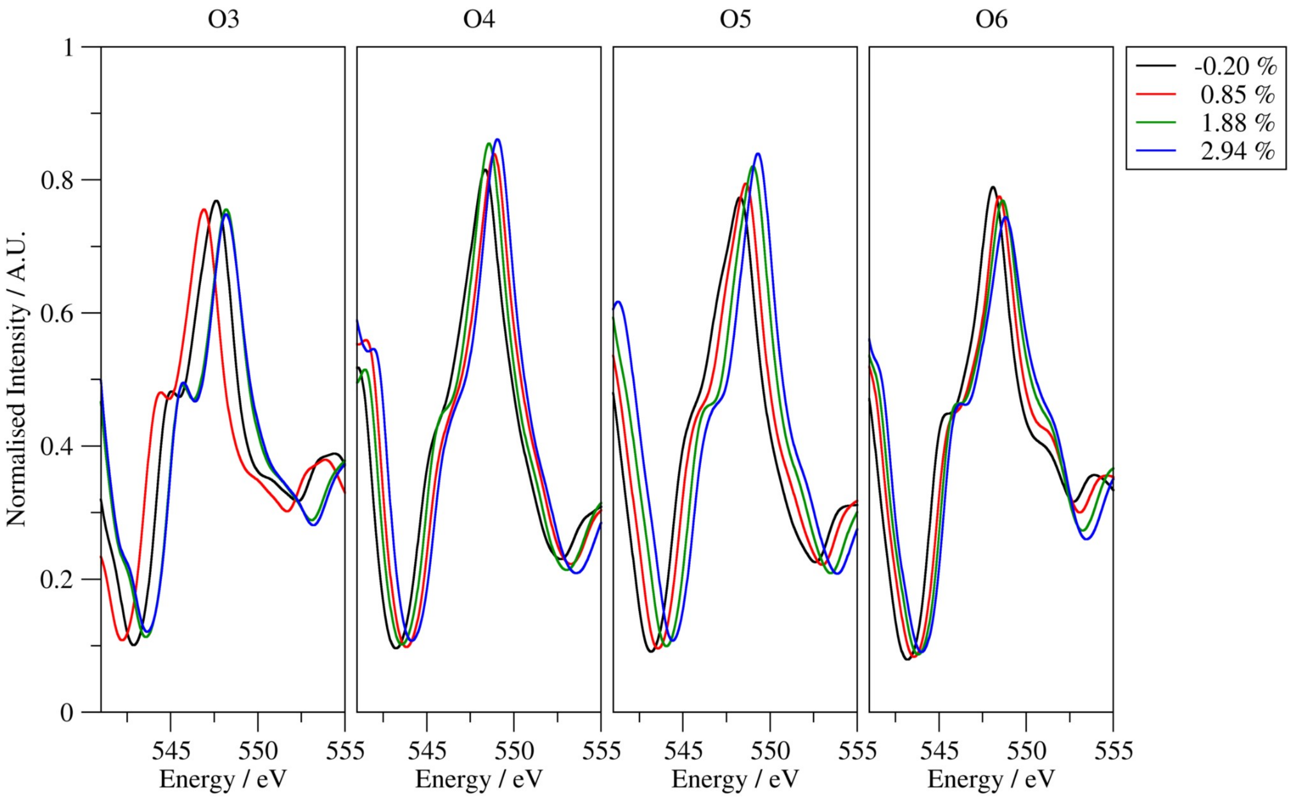

Figure S21. B peaks for the equatorial near Mn case for the 50% Mn doped system.

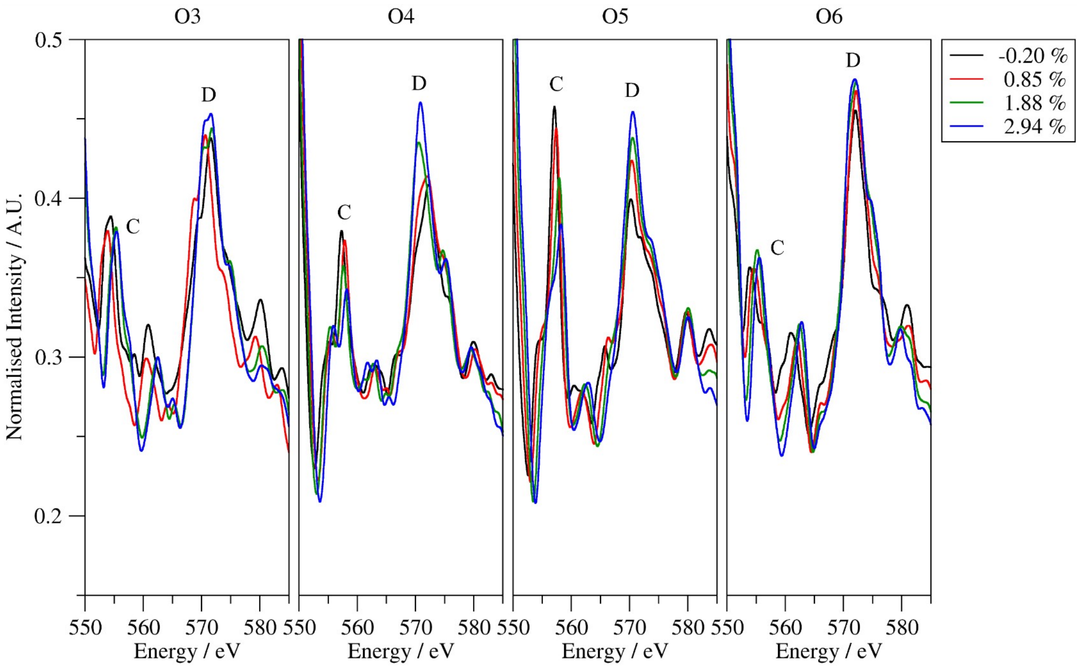

Figure S22. C and D peaks for the equatorial near Mn case for the 50% Mn doped system.

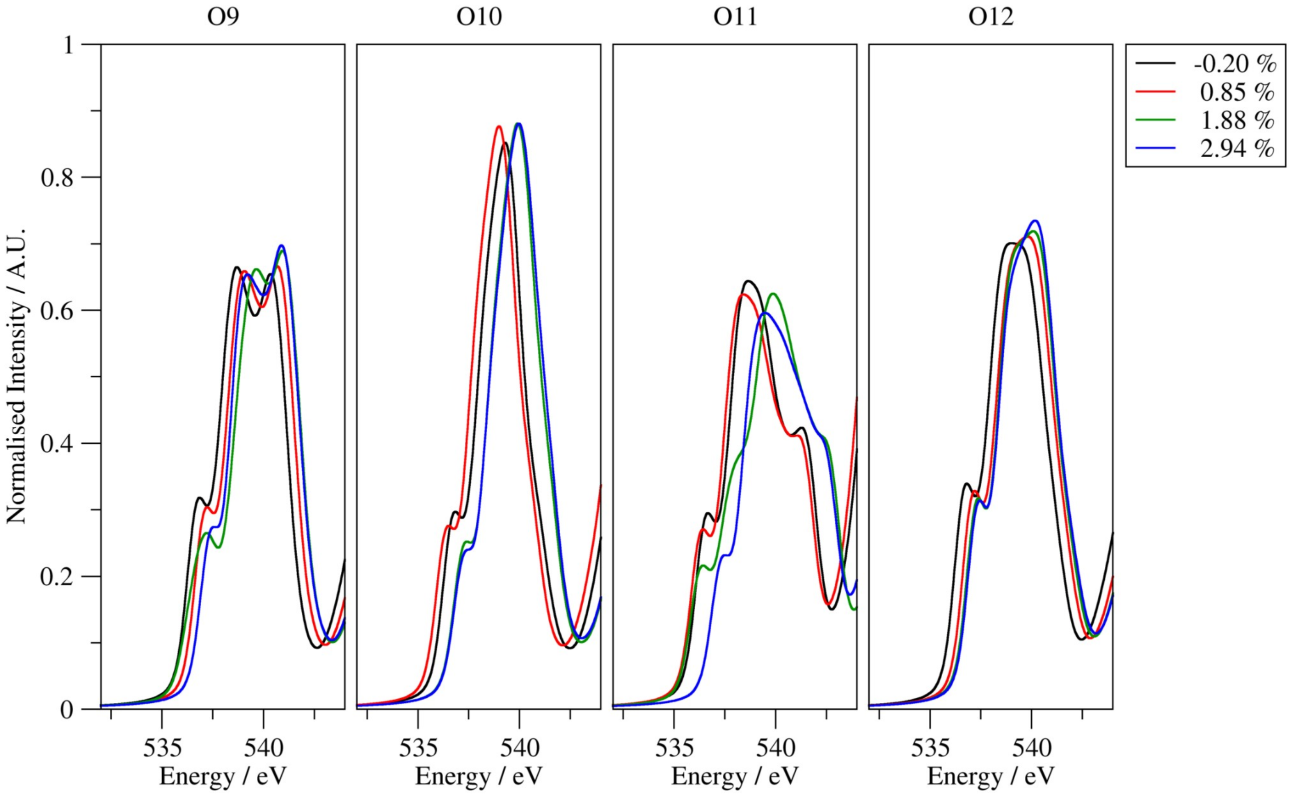

Figure S23. P and A peaks for the equatorial not near Mn case for the 50% Mn doped system.

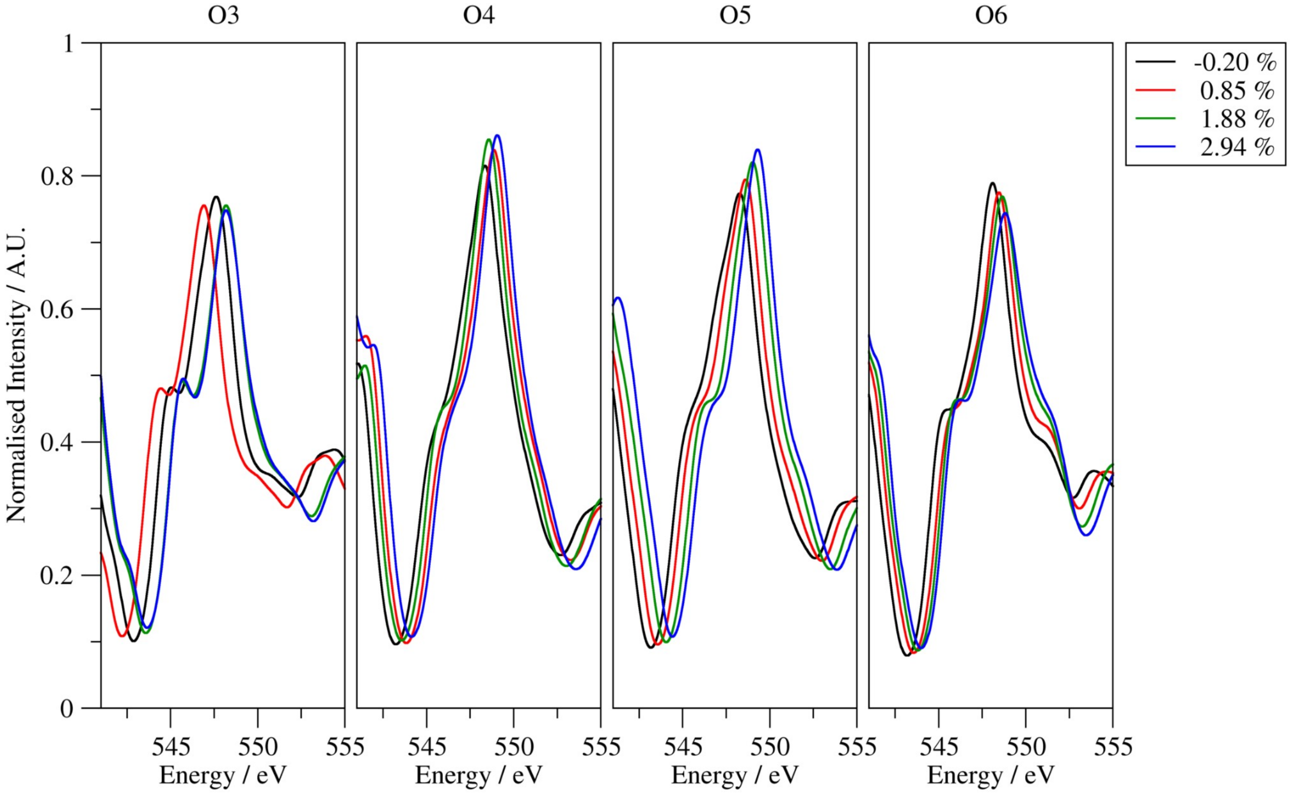

Figure S24. B peaks for the equatorial not near Mn case for the 50% Mn doped system.

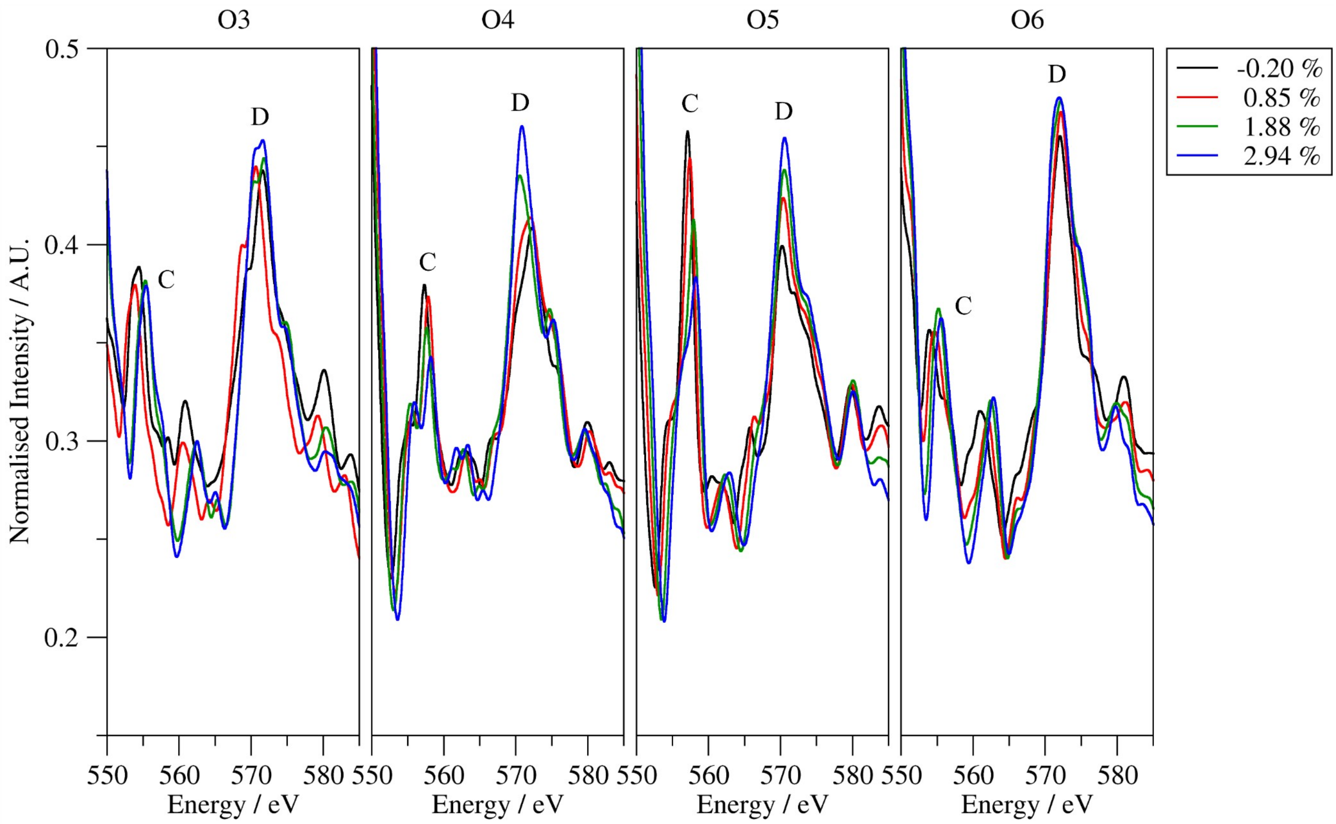

Figure S25.C and D peaks for the equatorial not near Mn case for the 50% Mn doped system.
